# Evidences of a Direct Relationship between Cellular Fuel Supply and Ciliogenesis Regulated by Hypoxic VDAC1-ΔC

**DOI:** 10.3390/cancers12113484

**Published:** 2020-11-23

**Authors:** Monique Meyenberg Cunha-de Padua, Lucilla Fabbri, Maeva Dufies, Sandra Lacas-Gervais, Julie Contenti, Charles Voyton, Sofia Fazio, Marie Irondelle, Baharia Mograbi, Matthieu Rouleau, Nirvana Sadaghianloo, Amandine Rovini, Catherine Brenner, William J. Craigen, Jérôme Bourgeais, Olivier Herault, Frédéric Bost, Nathalie M. Mazure

**Affiliations:** 1 INSERM U1065, University of Côte d’Azur (UCA), C3M, 151 Route de St Antoine de Ginestière, BP2 3194, CEDEX 03, 06204 Nice, France; monique.padua@sistemafiep.org.br (M.M.C.-d.P.); lucilla.fabbri@unice.fr (L.F.); contenti.j@chu-nice.fr (J.C.); charles.voyton@univ-cotedazur.fr (C.V.); sofia.fazio@univ-cotedazur.fr (S.F.); marie.irondelle@univ-cotedazur.fr (M.I.); sadaghianloo.n@chu-nice.fr (N.S.); frederic.bost@unice.fr (F.B.); 2 Medical Biology Department, Centre Scientifique de Monaco (CSM), 98000 Monaco, Monaco; maeva.dufies@unice.fr; 3 Centre Commun de Microscopie Appliquée, University of Côte d’Azur (UCA), 06000 Nice, France; sandra.lacas-gervais@unice.fr; 4 Department of Emergency Medicine, Centre Hospitalier Universitaire de Nice, 06001 Nice, France; 5 Centre Antoine Lacassagne, CNRS-UMR 7284-Inserm U1081, University of Côte d’Azur (UCA), 33 Ave. de Valombrose, 06189 Nice, France; baharia.mograbi@unice.fr; 6 LP2M, CNRS-UMR 7370, Faculty of Medicine, University of Côte d’Azur (UCA), 06108 Nice, France; rouleau@unice.fr; 7 Department of Vascular Surgery, Centre Hospitalier Universitaire de Nice, 06001 Nice, France; 8 Drug Discovery & Biomedical Sciences, Medical University of South Carolina, Charleston, SC 29425, USA; rovini@musc.edu; 9 Centre National de Recherche Scientifique (CNRS), Aspects Métaboliques et Systémiques de L’oncogénèse pour de Nouvelles Approches Thérapeutiques, Université Paris-Saclay, 94805 Villejuif, France; catherine.brenner@universite-paris-saclay.fr; 10 The Mitochondrial Diagnostic Laboratory, Department of Molecular and Human Genetics, Baylor College of Medicine, Houston, TX 77030, USA; wcraigen@bcm.edu; 11 CNRS ERL7001 LNOX «Leukemic Niche and Redox Metabolism», EA 7501 Unversité de Tours—CHU de Tours, Service d’Hématologie Biologique, 37032 Tours, France; j.bourgeais@chu-tours.fr (J.B.); olivier.herault@univ-tours.fr (O.H.); 12 CNRS GDR 3697 MicroNiT “Microenvironment of Tumor Niches”, 37032 Tours, France

**Keywords:** ciliopathy, glycolysis, mitochondrial respiration, primary cilium, tubulin, voltage-dependent anion channel 1

## Abstract

**Simple Summary:**

Here, we demonstrate that the hypoxia-induced cleaved form of VDAC1 (VDAC1-ΔC) reprograms the cell to utilize more metabolites and is implicated in up-regulation of glycolysis and mitochondrial respiration, conferring a direct survival advantage in hypoxic microenvironment. We further highlight a direct relationship between VDAC1-ΔC, the primary cilium and cell metabolism.

**Abstract:**

Metabolic flexibility is the ability of a cell to adapt its metabolism to changes in its surrounding environment. Such adaptability, combined with apoptosis resistance provides cancer cells with a survival advantage. Mitochondrial voltage-dependent anion channel 1 (VDAC1) has been defined as a metabolic checkpoint at the crossroad of these two processes. Here, we show that the hypoxia-induced cleaved form of VDAC1 (VDAC1-ΔC) is implicated in both the up-regulation of glycolysis and the mitochondrial respiration. We demonstrate that VDAC1-ΔC, due to the loss of the putative phosphorylation site at serine 215, concomitantly with the loss of interaction with tubulin and microtubules, reprograms the cell to utilize more metabolites, favoring cell growth in hypoxic microenvironment. We further found that VDAC1-ΔC represses ciliogenesis and thus participates in ciliopathy, a group of genetic disorders involving dysfunctional primary cilium. Cancer, although not representing a ciliopathy, is tightly linked to cilia. Moreover, we highlight, for the first time, a direct relationship between the cilium and cancer cell metabolism. Our study provides the first new comprehensive molecular-level model centered on VDAC1-ΔC integrating metabolic flexibility, ciliogenesis, and enhanced survival in a hypoxic microenvironment.

## 1. Introduction

Metabolic flexibility in cancer is a survival process allowing tumor cells to adapt to changes caused by hyper proliferation, glucose deprivation, systemic factors released by tumors, oxygen gradient, or anti-cancer treatments. Thus, understanding the underlying mechanisms of metabolic flexibility is crucial and has been intensively studied during the past twenty years [1,2]. Accordingly, new strategies for exploiting metabolic differences between normal and neoplastic cells are constantly being explored for therapy of cancer. However, the role mitochondria play in the context of hypoxia and how these organelles impact metabolism in oxygen starvation is still elusive.

Voltage-dependent anion channel 1 (VDAC1), the most abundant protein expressed at the outer membrane of the mitochondria (OMM), has been shown to be at the crossroads of metabolism and cell death [3,4]. As a mitochondrial pore, VDAC1 allows passage of important metabolites including ATP, NAD+, and ions between mitochondria and cytoplasm. VDAC1 also serves as an anchoring platform for critical metabolic proteins including Hexokinase, the first hypoxia-induced glycolytic enzyme, or Bcl-2 family proteins thus implicating a role in cell death by apoptosis [5,6]. Any modification affecting the protein will then significantly impact metabolism, cell growth or both. We observed a new form of VDAC1, VDAC1-∆C, in hypoxic conditions, characterized by a cleavage resulting in the loss of its C-terminus tail (CTT) [7]. VDAC1 is cleaved via the asparagine endopeptidase, Legumain (LGMN), which specifically cleaves VDAC1 at asparagine 214 [8]. This process requires HIF-1 and p53wt and is associated with the presence of enlarged mitochondria. Tumor cells expressing VDAC1 and containing VDAC1-ΔC had an upregulation of metabolism (both oxidative phosphorylation (OXPHOS) and glycolysis) and were resistant to apoptosis driven by staurosporine or etoposide [7]. We then demonstrated that knockout of Vdac1 expressing oncogenic RAS in murine embryonic fibroblasts (MEFs) potentiates tumor development in mice by promoting metabolic reprogramming, accelerating vascular destabilization and inflammation [9]. Rostovtseva et al. [10,11] showed that the pore formed by VDAC can be regulated by dimeric tubulin, one of the most abundant cytoskeleton proteins, having fateful repercussions on the metabolic cell responses [12]. The interaction of tubulin, specifically the anionic CTT of tubulin, acts as a plug within the VDAC pore, which induces VDAC closure, thus restraining mitochondrial respiration.

Although VDACs are mainly localized at the OMM, other subcellular locations have been reported, suggesting their implication in other processes. Recently, our data described a new link between ciliogenesis and VDAC1-ΔC [13]. In parallel, a new function has been described for VDAC3 in regulating the centriole assembly, therefore triggering inappropriate ciliogenesis [14]. In addition, Majunder et al. [15] showed that centrosomal VDAC1, not expressed at the mitochondrial compartment, negatively regulated ciliogenesis, similar to VDAC3. VDAC1 was found strongly localized at one of the centrioles, mainly associated with the mother centriole, likely mediated by its binding directly to microtubules, or a microtubule binding protein.

In the present study, by targeted mutation of VDAC1-ΔC in Ras MEFs followed by real time analysis of metabolism, transmission electron microscopy, metabolomics, and survival analysis, we identify a new metabolic function of mitochondrial VDAC1-ΔC controlling metabolic flexibility and the microtubule-enriched primary cilium assembly in hypoxia.

## 2. Materials and Methods

### 2.1. Cell Culture

RASV12-transformed mouse embryonic fibroblasts (Ras MEFs) cells were grown in Dulbecco’s Modified Eagle’s Medium (DMEM) (Gibco-BRL, Courtaboeuf, France) supplemented with 10% fetal bovine serum with penicillin G (50 U/mL) and streptomycin sulfate (50 µg/mL).

An INVIVO2 200 anaerobic workstation (Ruskinn Technology Biotrace International Plc, The Science Park Bridgend, UK) set at 1% oxygen, 94% nitrogen and 5% carbon dioxide was used for hypoxic conditions.

#### 2.1.1. Pharmacological Inhibitors and Chemicals

Cells were incubated with 1 µM staurosporine (STS) to induce cell death, 17 µM chloroquine (CQ) to block autophagy, 10 µM taxol (Taxol10) to stabilize the microtubule cytoskeleton, 1 µM colchicine (Colc.1) to inhibit polymerization of microtubules, and 50 µM vismodegib to inhibit the Hedgehog (Hh) signal transduction pathway. Rotenone, antimycin A, oligomycin, and trifluorocarbonylcyanide phenylhydrazone (FCCP) were from Sigma, St. Louis, MI, USA).

#### 2.1.2. RNA Interference

The 21-nucleotide siVDAC3 was from Mission esiRNA Sigma.

#### 2.1.3. Mutations and Stable Transfections

Valine (V) substitution for glycine (G) and glutamine (Q) for asparagine (N) at amino acid positions 213 and 214, respectively, in human VDAC1 was performed following the procedure according to the QuickChange II site-Direct Mutagenesis kit (Agilent, Les Ulis, France): plasmid pcDNA expressing the human VDAC1 protein was a gift from T. Rudel [16]. For multiple-site mutations, the PCR reaction of 50 μL contained 50 ng of template, 125 ng of primer pair, 200 μM dNTPs, and 2.5 units of Pfu DNA polymerase. The PCR cycles were initiated at 95 °C for 5 min to denature the template DNA, followed by 18 amplification cycles. Mutation verifications were carried out.

Vdac1 null (Vdac1^−/−^) RASV12-transformed mouse embryonic fibroblasts (Ras MEFs) cells were co-transfected with pVDAC1wild-type (Wt) or pVDAC1mutated213-214 (VDAC1MUT) and pBabepuro. Puromycin-resistant clones were tested for their ability to stably overexpress both Wt (Cleavable#1 and #2) and VDAC1MUT (UnCleavable#1 and #2) in normoxia and for their ability to express VDAC1-ΔC in hypoxia only in the Wt.

#### 2.1.4. Colony-Forming Assay

Cells (5000–10,000) were plated on 60-mm dishes and incubated at 37 °C, 5% CO_2_ for colony formation. After 10 days, colonies were fixed with 10% (*v*/*v*) methanol for 15 min and stained with 5% Giemsa (Sigma, St. Louis, USA) for 30 min for colony visualization.

#### 2.1.5. Respirometry and Extracellular Acidification

The cellular oxygen consumption rate (OCR) and extracellular acidification rate (ECAR) were obtained using a Seahorse XF24 extracellular flux analyzer from Seahorse Bioscience (North Billerica, MA, USA). Experiments were performed according to the manufacturer’s instructions. OCR and ECAR were measured in real time in normoxia or hypoxia. Cells were deprived of glucose for 1 h, then glucose (G–10 mM), oligomycin (O–1 µM), FCCP (F–3 µM), and Rotenone + Antimycin A (R/A–1 µM) were injected at the indicated times. Protein standardization was performed after each experiment, with no noticeable differences in protein concentration and cell phenotype.

#### 2.1.6. Phenotype MicroArray on Omnilog™ Analyser

Metabolic profiling was studied by using the Omnilog^®^ Phenotype Microarray™ system (Biolog, Hayward, CA, USA) evaluating the cell’s ability to metabolize 367 substrates. Cells were cultured for 48 h in normoxia or hypoxia, then transferred at seeding densities of 20,000 cells/well to the PM-M1 to 4 plates in a phenol red-free RPMI-1640-based medium depleted of carbon energy sources (IFM1 medium, Biolog Inc., Hayward, CA, USA), supplemented with 0.3 mM glutamine, 5% FCS, 100 U/mL penicillin, and 100 µg/mL streptomycin. Cells were then incubated for 24 h at 37 °C under 5% CO_2_ in hypoxia or normoxia before adding Biolog Redox Dye Mix MA, sealing the plate with tape to prevent gas transfer, and incubated at 37 °C in the Omnilog^®^ automated incubator-reader (Biolog Inc., Hayward, CA, USA) for 24 h to kinetically measure tetrazolium reduction resulting in formation of a purple color. Data was collected on a PMM Kinetics software with subtraction of the average values of three negative control wells (background), then, analysis was computed with R software (Version 3.4.4) with the opm package.

#### 2.1.7. ATP Determination

RAS MEF (Wt, Vdac1^−/−^, Vdac1^−/−^ + VDAC#1, Vdac1^−/−^ + VDAC#2, Vdac1^−/−^ + VDAC MUT#1, Vdac1^−/−^ + VDAC MUT#2) were incubated in hypoxia for 72 h and then lysed. ATP was quantified using a luciferin/luciferase-based assay (Cell Titer Glo kit, Promega, Madison, WI, USA) according to the manufacturer’s instructions and results are expressed as relative luminescence units (RLU). Each condition was tested eight times and the entire experiment was done twice.

#### 2.1.8. Lactate Measurement

The lactate concentration in the supernatant of cells incubated either in normoxia or hypoxia for 48 h was determined by an enzyme-based assay using 900 µM β-NAD (BioChemika, St. Quentin Fallavier, France), 175 µg/mL L-lactate dehydrogenase (BioChemika, St. Quentin Fallavier, France), and 100 µg/mL glutamate–pyruvate transaminase (Roche) and was diluted in a sodium carbonate (620 mM) L-glutamate (79 mM) buffer adjusted to pH 10. Lithium lactate was used as a standard. Measurements were done with a microplate reader after incubation for 30 min at 37 °C. For each condition, the protein concentration was determined to express the lactate concentration as mmol/µg protein.

#### 2.1.9. FACS Analysis

For determination of the cell cycle in RAS MEF (Wt, Vdac1^−/−^, Cleavable#1, Cleavable#2, UnCleavable#1, UnCleavable#2), cellular suspensions (5 × 10^5^ cells) were resuspended in HBSS + FBS 10% and rinsed 3 times in cold PBS. Then, the cells were fixed with Ethanol 70% at 4 °C. Cell suspensions were then incubated with Propidium iodide solution 1 mg/mL (Sigma-Aldrich) and RNAase (Life Technologies, Carlsbad, CA, USA) for 30 min at 4 °C. Samples were collected with Miltenyi MCSQuant10 (Bergisch Gladbach, Germany) and analyzed with FlowJo Software (Version 10.6).

#### 2.1.10. Microtubule Assay

The in vitro assay that identified proteins that bind to microtubules (MT) was performed as prescribed by the manufacturer (Cytoskeleton, Denver, CO, USA). The microtubule associated protein (MAP) fraction was used as a positive control and bovine serum albumin (BSA) was used as negative control.

#### 2.1.11. Electron Microscopy

For ultrastructural analysis, cells were fixed in 1.6% glutaraldehyde in 0.1 M phosphate buffer pH7.3, rinsed in 0.1 M cacodylate buffer, post-fixed for 1h in 1% osmium tetroxide and 1% potassium ferrocyanide in 0.1 M cacodylate buffer to enhance the staining of membranes. Cells were rinsed in distilled water, dehydrated in alcohol, and lastly embedded in epoxy resin. Contrasted ultrathin sections (70 nm) were analyzed under a JEOL 1400 transmission electron microscope mounted with a Morada Olympus CCD camera.

### 2.2. Immunoblotting

Cells were lysed in 1.5× SDS buffer and the protein concentration determined using the BCA assay; 40 µg of protein from whole cell extracts were resolved by SDS-PAGE and transferred onto a PVDF membrane (Millipore, Molsheim, France). Membranes were blocked in 5% non-fat milk in TN buffer (50 mM Tris-HCl pH 7.4, 150 mM NaCl) and incubated in the presence of the primary and then secondary antibodies in 5% non-fat milk in TN buffer. The rabbit polyclonal antibody to central regions of VDAC1 was purchased from Abcam, Cambridge, UK (ab15895). Anti-LC3 was raised in rabbits immunized against the N-terminal 14 amino acids of human LC3 and was produced and characterized in our laboratory [17]. Mouse anti-acetylated tubulin (T7451), anti-β-tubulin, anti-α-tubulin, and β-actin were from Sigma, St. Louis, USA. ECL signals were normalized to either β-tubulin or ARD1 [18]. After washing in TN buffer containing 1% Triton-X100 and then in TN buffer, immunoreactive bands were visualized with the ECL system (Amersham Biosciences, Buckinghamshire, UK).

#### Co-Immunoprecipitation Assay

Cells (2 × 10^6^) were lysed in RIPA-TRIS EDTA (20 mM Tris-HCl (pH7.5), 150 mM NaCl, 1 mM Na2 EDTA, 1 mM EGTA, 1% NP-40, 1% sodium deoxycholate, 2.5 mM sodium pyrophosphate, 1 mM β-glycerophosphate, 1mM Na3VO4, 1 µg/mL leupeptin) buffer, and the protein concentration determined using the BCA assay; 500 µg of protein from whole cell extracts were used and incubated with β-III Tubulin (D71G9—Cell signaling, Danvers, MA, USA) plus DynaBeads Protein G (Invitrogen, Carlsbad, CA, USA) overnight. The following day, the immunoblotting against VDAC-1 or Tubulin β-III was performed as mentioned before. The IgG from rabbit was used as negative control.

### 2.3. Immunocytochemistry

Cells were fixed in 3% paraformaldehyde and extracted with Triton X-100. Primary antibodies included mouse anti-acetylated tubulin (Sigma-Aldrich, Basel, Switzerland) (1:400). Alexa Fluor 594- and 488-conjugated secondary goat anti-mouse or goat anti-rabbit antibodies (Molecular Probes, Carlsbad, CA, USA) were used at 1:400. Cells were visualized by wide-field, fluorescence microscopy using a DM5500B upright stand (Leica, Wetzlar, Germany) with a 40× oil objective NA 1.00. The cubes used were A4 (excitation filter BP 360/40, dichroic mirror 400, emission filter BP 470/40), L5 (BP 480/40, 505, BP 527/30), and TX2 (BP 560/40, 595, BP645/75). Acquisitions were done with an Orca-ER camera (Hamamatsu, Hamamatsu, Japan). Cells were also visualized using the confocal microscope, Axiovert 200 M inverted stand (Zeiss, Oberkochen, Germany). Objectives 10× dry NA 0.3 and/or 25× multi immersion (oil, glycerol, water) NA 0.75, and/or 40× oil 1.3 NA and/or 63× oil 1.4 NA were used. The LASER used were diode 405 nm, and/or Argon 488 nm, and/or HeNe 543 nm. The microscope was equipped with an automated xy stage for mosaic acquisitions.

Cilia frequency was counted manually from scans using a 40× digital zoom for 100–300 nuclei.

#### 2.3.1. Microarray Experiments

Microarray experiments were already described [9]. The experimental data have been deposited on the NCBI Gene Expression Omnibus (GEO) (http://www.ncbi.nlm.nih.gov/geo/) under the series record number GSE63247.

#### 2.3.2. Statistics

All values are the means ± SEM. Statistical analyses were performed using the Student’s *t*-test in Microsoft Excel. The *p* values are indicated. All categorical data used numbers and percentages. Quantitative data are presented using the median and range or mean. Differences between groups were evaluated using the chi square test for categorical variables and the Student’s *t*-test for continuous variables. Analyses were performed using SPSS 16.0 statistical software (SPSS Inc., Chicago, IL, USA). All statistical tests were two-sided, and *p*-values < 0.05 indicated statistical significance, whereas *p*-values between 0.05 and 0.10 indicated a statistical tendency.

## 3. Results

### 3.1. Genetic Proof of Concept in Cellular Model

**Figure 1:**
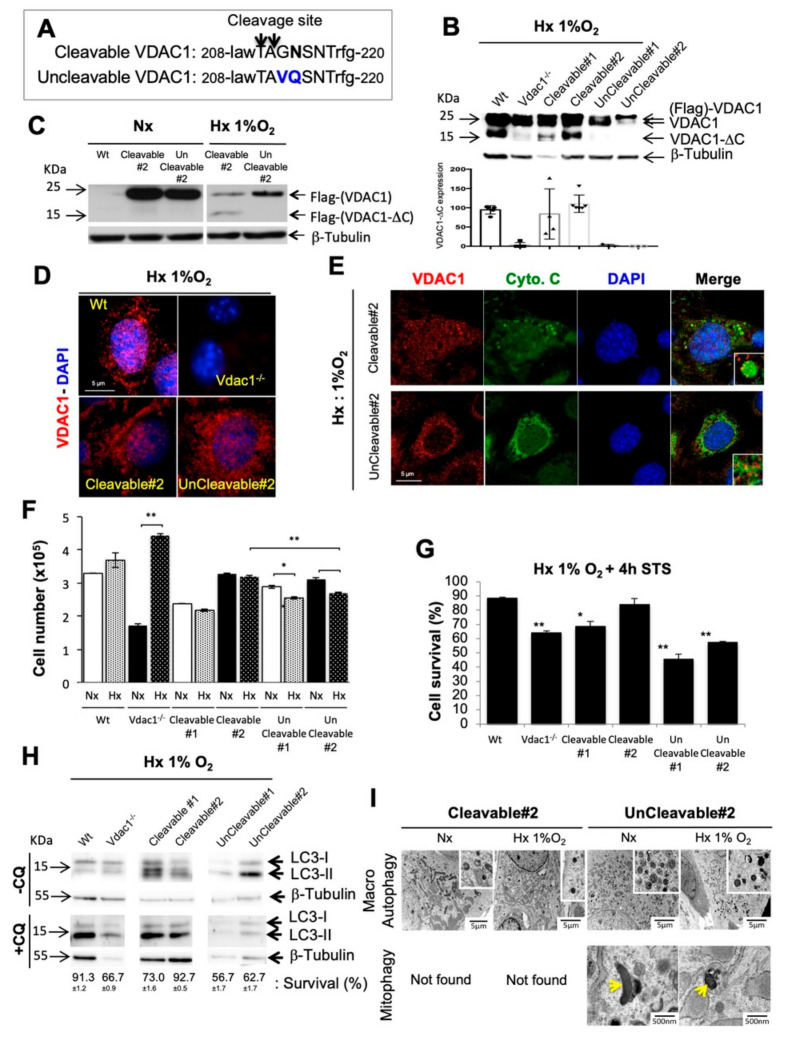
Characterization of cleavable and uncleavable clones of Voltage-dependent anion channel 1 (VDAC1) in normoxia and hypoxia. (**A**) Schematic representation of mutations made at the cleavage site of VDAC1. (**B**) Wt, Vdac1^−/−^, Cleavable#1/#2 and UnCleavable#1/#2 cells were incubated in Hx 1% O_2_ for 72 h and cell lysates were analyzed by immunoblotting for VDAC1. β-tubulin was used as a loading control. Bottom panel showed quantification of VDAC1-∆C presence in at least four different immunoblots. (**C**) *Vdac1*^−/−^, Cleavable #2 and UnCleavable #2 cells were incubated in Hx 1% O_2_ for 72 h and cell lysates were analyzed by immunoblotting for Flag (mouse monoclonal antibody). β-tubulin was used as a loading control. (**D**) Immunofluorescence of VDAC1 in Wt, Vdac1^−/−^, Cleavable#2 and UnCleavable#2 cells in Hx 1% O_2_ for 72 h. (**E**) Immunofluorescence to VDAC1 and cytochrome c (Cyto.C) in Cleavable#2 and UnCleavable#2 cells in Hx 1% O_2_ for 72 h. (**F**) Cell lines were seeded at the same density and incubated in Nx or Hx 1% O_2_ for 72 h. The mean ± SEM is representative of four independent experiments carried out in duplicate. (**G**) Wt, Vdac1^−/−^, Cleavable#1/#2 and UnCleavable#1/#2 cells were incubated in Hx 1% O_2_ for 72 h and challenged with staurosporin (STS) (1 µM) for 4 h. Cell mortality was measured using an ADAM cell counter. (**H**) Wt, Vdac1^−/−^, Cleavable#1/#2 and UnCleavable#1/#2 cells were incubated in Hx 1% O_2_ for 72 h in the presence of chloroquine (CQ) and cell lysates were analyzed by immunoblotting for LC3-I/II. β-tubulin was used as a loading control. Cell viability was measured using an ADAM cell counter. The mean ± SEM is representative of three independent experiments carried out in duplicate. (**I**) Representative electron micrographs of mitochondria from Cleavable#2 and UnCleavable#2 cells incubated in normoxia (Nx) or Hx 1% O_2_ for 72 h. Yellow arrows show mitochondria engulfed in autophagosomes/autolysosomes. A * *p* < 0.05 and ** *p* < 0.005 show significant differences. The uncropped Western Blot figures in Figure S10.

To set up an instrumentable cellular model, we modeled non-cleavable VDAC1 in hypoxia via the addition of multiple-site directed mutants of human VDAC1 without a LGMN cleavage domain [8]: position 213 (Gly to Val) and 214 (Asp to Gln) (Figure 1A). Plasmids expressing wild-type VDAC1 (Wt) [16] and mutant VDAC1 were stably transfected into Vdac1 null (Vdac1^−/−^) RasV12-transformed mouse embryonic fibroblasts (Ras MEFs) [9]. Two clones expressing wild-type cleavable VDAC1 (Cleavable#1/#2) Ras MEFs and two clones expressing a mutated non-cleavable VDAC1 (UnCleavable#1/2) Ras MEFs clones were obtained and grown in normoxia and hypoxia (Figure 1B–D and Figure S1A). Cleavable#1 cells were chosen as they express a lower amount of wild-type cleavable VDAC1 compared to Wt cells and Cleavable#2 cells, as they expressed a similar amount of wild-type cleavable VDAC1 compared to Wt (Figure 1B). Cleavable#2 cells exposed to hypoxia containing VDAC1-ΔC, showed enlarged mitochondria, and exhibited growth rates identical to that of Wt cells (Figure 1E,F and Figure S1B). Cleavable#1 cells were similar (enlarged mitochondria) to Wt cells but to a lesser extent (lower proliferation). Hypoxic UnCleavable#1/#2 cells not containing VDAC1-ΔC, showed a tubular mitochondrial network and a significant decrease in proliferation compared to Cleavable#2.

To examine resistance to intrinsic death stimuli, cells were incubated with staurosporine (STS). Expression of VDAC1-ΔC significantly inhibited apoptosis in Wt (88.5% ± 0.7) of cell survival), Cleavable#2 (84% ± 3.5) cells in hypoxia after 72 h (Figure 1G) or 8 d (Figure S1C). Cleavable#1 (68.5%) cells which express less VDAC1-ΔC, showed intermediate apoptosis level. In contrast, Vdac1^−/−^ (64%) and UnCleavable#1/#2 (45.5% ± 3.5 and 57.5% ± 0.7, respectively) cells responded to STS. As VDACs served as mitochondrial docking sites to promote mitophagy [19], we investigated the direct role of VDAC1-ΔC on both autophagy and mitophagy. A higher basal level of autophagy was observed in UnCleavable#2 cells compared to the Cleavable#2 control in hypoxia (Figure 1H). Blocking the autophagic flux with chloroquine (CQ), a lysosomotropic agent that prevents endosomal acidification, did not impact cell survival for Wt, Cleavable#1/#2 cells, whereas Vdac1^−/−^, UnCleavable#1/#2 cells showed a drastic reduction in survival. Tubular mitochondria engulfed in autophagosomes/autolysosomes were characterized only in UnCleavable#2 cells in normoxia and hypoxia, suggesting that mitophagy was a basal process in these cells and not in the Cleavable#2 cells (Figure 1I). However, no mitophagy was found in Cleavable#2 cells, neither in normoxia or hypoxia.

These results show that hypoxic cells, which were devoid of VDAC1-ΔC, kept the tubular mitochondria morphology, were sensitive to apoptosis, and relied on autophagy and mitophagy for survival. Moreover, the involvement of cells in these different processes is dependent on the amount of mitochondrial VDAC1-ΔC present in the cells. Therefore, these results support the survival functions of VDAC1-ΔC in Ras-transformed fibroblasts.

### 3.2. Metabolic Adaptability is Controlled by VDAC1-ΔC

**Figure 2:**
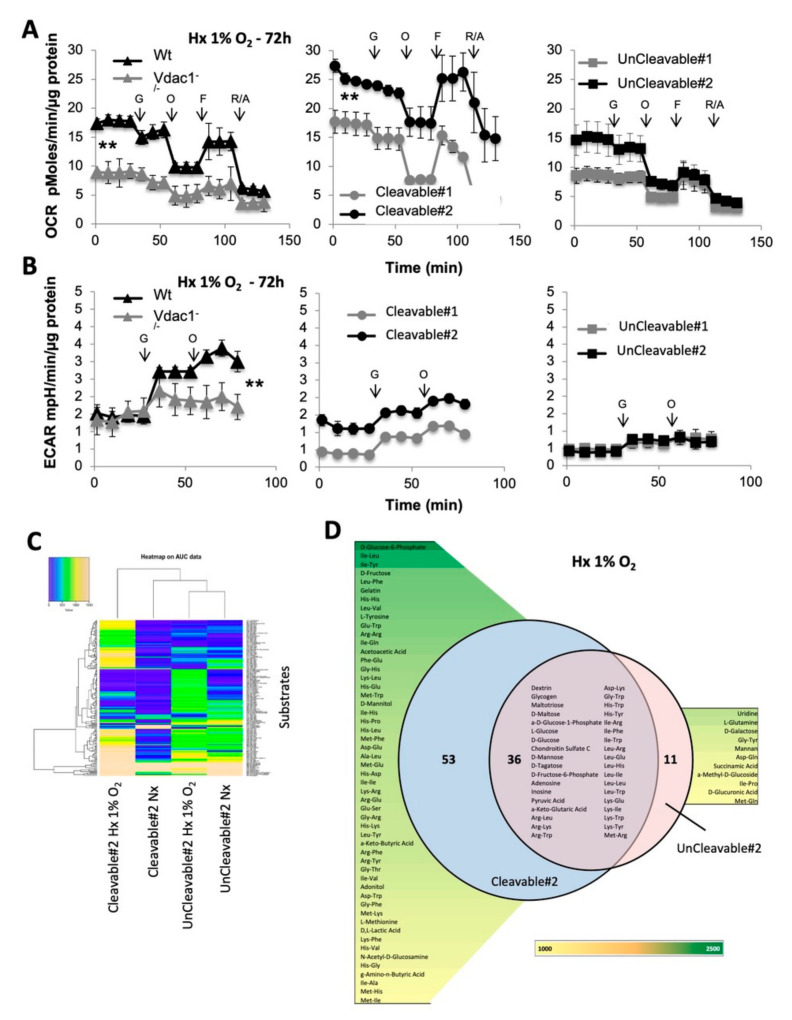
Metabolic characteristics of the cleavable and uncleavable forms of VDAC1 in 1% O_2_ hypoxia. (**A**) Respiratory control of Wt and Vdac1^−/−^ cells (left panel), Cleavable#1/#2 (middle panel) and UnCleavable#1/#2 (right panel). OCR was measured in real time with the XF24 analyzer in Hx 1% O_2_. Cells were deprived of glucose for 1h, then glucose (G), oligomycin (O), FCCP (F), and Rotenone + Antimycin A (R/A) were injected at the indicated times. The mean ± SEM is representative of at least three independent experiments carried out in quadruplicate. (**B**) ECAR in Hx of Wt and Vdac1^−/−^ cells (left panel), Cleavable#1/#2 (middle panel) and UnCleavable#1/#2 cells (right panel) was evaluated with the XF24 analyzer. Cells were deprived of glucose for 1 h, then glucose (G) and oligomycin (O) were injected at the indicated times. The mean ± SEM is representative of at least three independent experiments carried out in quadruplicate. (**C**) Heatmap showing the 160 substrates that were differently metabolized by Cleavable#2 and UnCleavable#2 in normoxia and hypoxia. The color key scale for each substrate is based on dye reduction quantified by Omnilog units. A yellow color indicates strong positive substrate metabolization, a green color moderate metabolization and a blue color indicates no substrate metabolization. Data area under the curve (AUC) <500 have been excluded. (**D**) Venn diagram comparing metabolic phenotyping of Cleavable#2 and UnCleavable#2 in hypoxia. Data AUC > 750. ** indicates *p* < 0.005.

As VDAC1 has been proposed to be a metabolic checkpoint between glycolysis and oxidative phosphorylation in the Warburg effect [10], known to affect most cancer cells, we first quantified mitochondrial respiration with the Seahorse XF by measuring the oxygen consumption rate (OCR) in Cleavable#1/#2 and UnCleavable#1/#2 cells compared to Wt and Vdac1^−/−^. Basal respiration, the rate of mitochondrial ATP synthesis and maximal respiration of Vdac1^−/−^and UnCleavable#1/#2 cells were lower versus Wt and Cleavable#2 cells in hypoxia after 72 h and 15 d (Figure 2A and Figure S2A). Cleavable#1 cells presented a respiration similar to Wt. Cleavable#2 cells presented a basal respiration 1.4-fold more active (24.13 ± 0.46 pMoles/min/µg g protein) and a higher respiration capacity (10.55 ± 3.8) compared to Wt (17.79 ± 0.8 pMoles/min/µg protein and 8.28 ± 0.47, respectively). However, no differences were observed in normoxia (Figure S3A). In parallel, the basal level of glycolysis and the maximal glycolytic capacity of Vdac1^−/−^ (1.59 ± 0.1 mpH/min/µg protein, 0.58 ± 0.1), UnCleavable#1/#2 cells (0.47 ± 0.12 mpH/min/µg protein, 0.35 ± 0.14; 0.39 ± 0.09 mpH/min/µg protein, 0.42 ± 0.2) were similar or lower than that of Wt (1.46 ± 0.18 mpH/min/µg protein, 1.93 ± 0.37), Cleavable#1/#2 cells (0.35 ± 0.02 mpH/min/µg protein, 0.84 ± 0.08; 1.11 ± 0.11 mpH/min/µg protein, 0.87 ± 0.13) in hypoxia after 72 h and 15 d (Figure 2B and Figure S2B). In contrast, Vdac1^−/−^ showed a higher glycolytic capacity compared to Wt cells under chronic hypoxia (15 d). Differences in glycolytic capacity were attenuated in normoxia (Figure S3B) but cells lacking VDAC1-ΔC still maintained a lower maximal glycolytic capacity. Relative to normoxia, lactate and ATP production were increased in Wt, Cleavable#1/#2 cells compared to Vdac1^−/−^, UnCleavable#1/#2 cells in hypoxia (Figure S4).

To gain an in-depth understanding of the magnitude and functional significance of the high metabolic capability of VDAC1-ΔC cells under hypoxic conditions, we evaluated different carbon energy substrate pathways from the OmniLog Phenotype MicroArray to obtain metabolic fingerprinting of Cleavable#2 and UnCleavable#2 cells in normoxia and hypoxia. 367 substrate nutrients were tested including carbohydrate and carboxylate substrates, individual L-amino acids and most dipeptide combinations.

In total, 89/367 (24.3%) and 47/367 (12.8%) substrates were metabolized by Cleavable#2 and UnCleavable#2 cells in hypoxia, respectively. The heatmap in Figure 2C shows preferential utilization of these 160 substrates. UnCleavable#2 cells in hypoxia compared to normoxia, showed less color key scale modification, shared similar metabolic profiles, which suggests less metabolic adaptation capacity in hypoxia. However, Cleavable#2 cells showed different metabolic patterns between normoxia and hypoxia with more substrates being metabolized in hypoxia. We compared the metabolic substrates used between the two clones: 53 substrates were used only by Cleavable#2 cells, 11 substrates by UnCleavable#2 cells and 39 were common across phenotypes (Figure 2D). As expected, both clones grew on glucose or glycogen as well as pyruvate. Additional metabolites were associated with both clones, including 21 different dipeptides among them Leucine (Leu) (×6) and Lysine (Lys) (×4) were present. However, a number of metabolites were uniquely associated with either Cleavable#2 cells or UnCleavable#2 cells. D-Glucose-6-Phosphate, D-Mannitol, D-Fructose, or D, L-Lactic Acid were associated with Cleavable#2 cells whereas Succinamic Acid, D-Glucuronic Acid and L-Glutamine were associated with UnCleavable#2 cells. Moreover, Cleavable#2 cells were associated with 42 dipeptides, where Histidine (His) (×8), Isoleucine (Ile) (×7) and Methionine (Met) (×6) residues were the most present, whereas UnCleavable#2 cells were associated only with 4 dipeptides (Figure S5A). We used the principle component analysis (PCA) to analyze our data differences and ascertain the main variables within a multidimensional data set. Cleavable#2-hypoxia clone clearly separated from the other clones across the first component confirming the highest modification in the metabolic phenotype (Figure S5B). A functional cartography of complex metabolic networks illustrates that on Figure S5C,D.

Collectively, these results support a model in which cells expressing both VDAC1 and VDAC1-ΔC grew better in hypoxia via maintenance of an increased rate of respiration, and promoting glycolysis compared to cells expressing the non-cleavable form of VDAC1, which indicates that the ratio between VDAC1 and VDAC1-ΔC is crucial to control the carbon metabolism. VDAC1-ΔC conferred significant metabolic advantages to Ras MEFs in hypoxia as they metabolize more carbon sources and dipeptides versus Ras MEFS with the non-cleavable VDAC1.

### 3.3. Mitochondrial VDAC1-ΔC Represses Ciliogenesis

**Figure 3:**
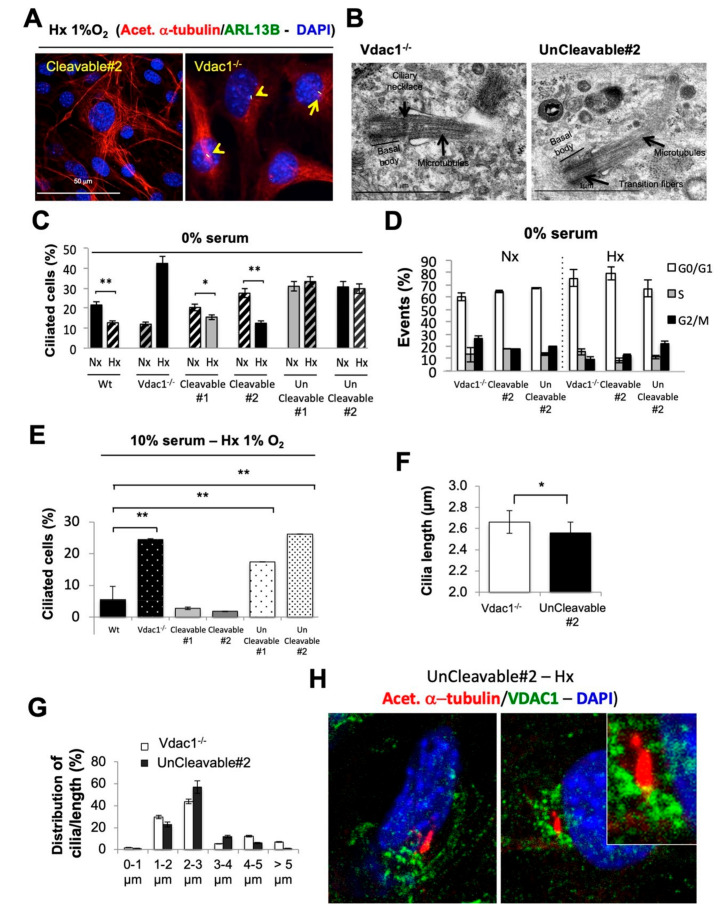
VDAC-ΔC drives the resorption of the primary cilium. (**A**) Immunofluorescence of acetylated α-tubulin (acet. α-tubulin in red), ARL13B (in green) and DAPI (in blue) in Cleavable#2 and *Vdac*1^−/−^ cells in Hx 1% O_2_ for 72 h. (**B**) Representative electron micrographs of primary cilia of UnCleavable#2 cells incubated in normoxia (Nx). Axoneme (Ax), basal body, ciliary necklace, microtubules and transition fibers. (**C**) Quantitative analysis of the effect of Nx and Hx 1% O_2_ for 72 h on the ciliation percentage in the absence of serum (0% serum) in Wt, Vdac1^−/−^, Cleavable#1/#2, and UnCleavable#1/#2 cells assessed by confocal fluorescence microscopy (*n* = 100–300 cells). (**D**) Wt, Vdac1^−/−^, Cleavable#1/#2 and UnCleavable#1/#2 cells were incubated in Nx or Hx 1% O_2_ for in the absence of serum (0% serum). Cell cycle analysis was assessed by FACS. (**E**) Quantitative analysis of the effect of Nx and Hx 1% O_2_ for 72 h on the ciliation percentage in the presence of 10% serum in Wt, Vdac1^−/−^, Cleavable#1/#2, and UnCleavable#1/#2 cells as assessed by confocal fluorescence microscopy (*n* = 100–300 cells). (**F**) Average cilium length (µm) in Vdac1^−/−^ (*n* = 100) and UnCleavable#2 (*n* = 58) cells was assessed by confocal fluorescence microscopy. (**G**) The distribution of the average cilium length (µm) in Vdac1^−/−^ (*n* = 100) and UnCleavable#2 (*n* = 58) cells was assessed by confocal fluorescence microscopy. (**H**) Double staining and merge presentation of acetylated α-tubulin (acet. α-tubulin in red), VDAC1 (in green) and DAPI (in blue) in UnCleavable#2 cells in Hx 1% O_2_ for 72 h. * indicates *p* < 0.05 and ** indicates *p* < 0.005.

We next investigated the link between VDAC1-ΔC and primary cilia by both immunofluorescence using antibodies that recognize acetylated α-tubulin in the axoneme and Arl13B specifically localized to cilia (Figure 3A) and transmission electron microscopy (Figure 3B). We observed the presence of more primary cilia in Vdac1^−/−^ and UnCleavable#2 (data not shown) cells, which express the non-cleavable form of VDAC1 in hypoxia in comparison to Cleavable#2 cells. The complex structure of the primary cilium was clearly visible (Figure 3B). We found that the percentage of ciliated cells was significantly lower in Wt, and Cleavable1/#2 cells in hypoxia than in Vdac1^−/−^ and UnCleavable#1/#2 cells in conditions where cell cycle was controlled and similar (0% serum—Figure 3C,D) or not controlled (10% serum—Figure 3E). Surprisingly, only Vdac1^−/−^ cells showed more primary cilia in hypoxia. We found no impact of the VDAC2 down regulation on cilia (data not shown), whereas VDAC3-targeted siRNA cells (Figure S6A) decreased the number of ciliated cells by 50% (Figure S6B), strongly suggesting that ciliogenesis in *Vdac1*^−/−^ cells is Vdac3-dependent. We identified a trend towards primary cilia being more elongated in Vdac1^−/−^ cells compared to UnCleavable#2 cells (Figure 3F,G). Finally, as shown in Figure 3H, double immunofluorescent staining of VDCA1 and acetylated α-tubulin in Uncleavable#2 cells showed that both proteins localized at the same or close area of the end of the primary cilium. These data further support a new mechanism of control through mitochondrial VDAC1 regarding the biogenesis of the primary cilium in hypoxia that can be rescued by VDAC3 but not VDAC2 isoform.

### 3.4. When VDAC1 Meets Tubulin to Control Primary Cilium and Metabolism

**Figure 4:**
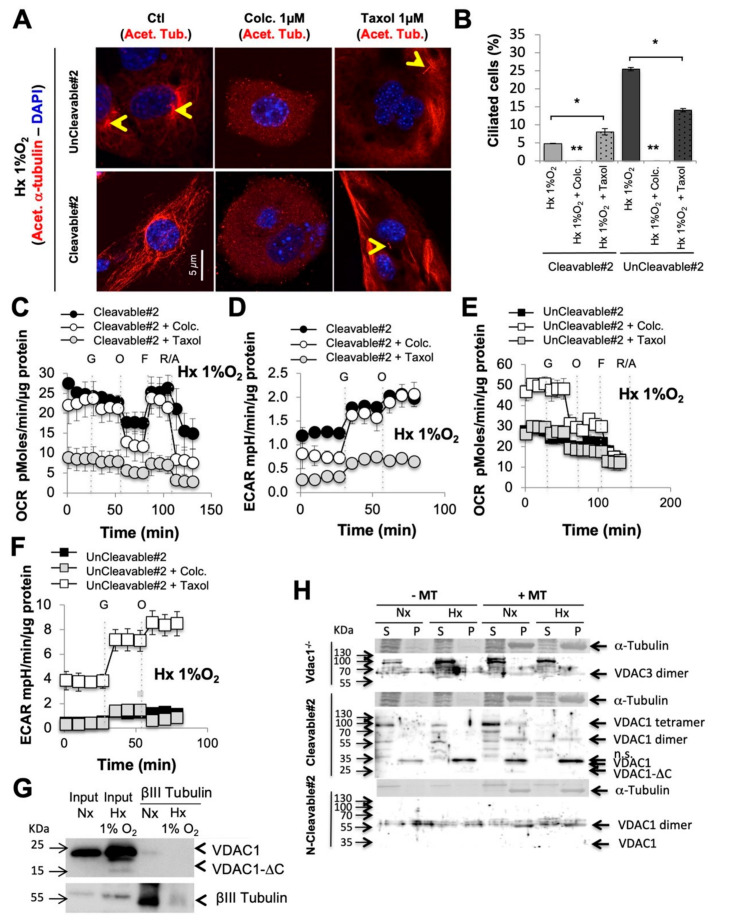
Destabilization or stabilization of the tubulin modified the percentage of primary cilium and metabolism, which was dependent on the cleaved form of VDAC1. (**A**) Immunofluorescence of acetylated α-tubulin (Acet. α-tubulin in red) and DAPI (in blue) in Cleavable#2 and UnCleavable#2 cells in the presence of colchicine (Colc.–1 µM) or taxol (1 µM) compared to the control (Ctl) in Hx 1% O_2_ for 72 h. (**B**) Quantitative analysis of the effect of the absence or presence of colchicine (Colc.–1 µM) or taxol (1 µM) for 72 h on the percentage of ciliation assessed by confocal fluorescence microscopy (*n* = 100–300 cells). (**C**) and (**D**) Respiratory control of Cleavable#2 (**C**) and UnCleavable#2 (**D**) in the presence of colchicine (Colc.) or taxol. OCR was measured in real time with the XF24 analyzer in Hx 1% O_2_. Cells were deprived of glucose for 1 h, then glucose (G), oligomycin (O), FCCP (F) and Rotenone + Antimycin A (R/A) were injected at the indicated times. (E) and (F) ECAR in Hx 1% O2 of Cleavable#2 (**E**) and UnCleavable#2 (**F**) in the presence of colchicine (Colc.) or taxol was evaluated with the XF24 analyzer. Cells were deprived of glucose for 1 h, then glucose (G) and oligomycin (O) were injected at the indicated times. (**G**) βIII Tubulin co-immunoprecipitating with VDAC1 in Wt cells in normoxia and hypoxia. (**H**) Pelleting assay for microtubule-associated proteins. Vdac1^−/−^, Cleavable#2 and UnCleavable#2 cells were incubated in the absence (−MT) or presence of taxol-stabilized microtubules (+MT). The amounts of VDAC1 and tubulin in the supernatant (S) and pellet (P) were analyzed by SDS-PAGE and immunoblotting. A * *p* < 0.05 and ** *p* < 0.005 show significant differences. The uncropped Western Blot figures in Figure S10.

The observed metabolic adaptation inferred by VDAC1/VDAC1-ΔC ratio prompted us to investigate the impact of the primary cilium on metabolism and vice versa using colchicine, which induces microtubule disassembly, and taxol, an antimitotic agent that inhibits microtubule depolymerization. Colchicine and taxol had a slight differential cytotoxic effect (Figure S7A) and slightly increased VDAC1 expression (Figure S7B). Colchicine destabilized tubulin (Figure 4A), which is evidenced by the observed decreased number of ciliated cells (Figure 4B), whereas taxol increased the percentage of primary cilia in Cleavable#2 cells but decreased it in UnCleavable#2 cells. A decreased percentage of primary cilia, in the presence of colchicine, did not affect mitochondrial respiration (Figure 4C) nor the glycolytic capacity in Cleavable#2 cells (Figure 4D) in hypoxia. However, reestablishment of the primary cilium, in the presence of taxol, in Cleavable#2 cells dropped both mitochondrial respiration and the glycolytic capacity. UnCleavable#2 cells respired more in the presence of colchicine (Figure 4E) and their glycolytic capacity was increased (Figure 4F). In addition, we tested vismodegib, a hedgehog signaling pathway inhibitor. Vismodegib acted similarly to colchicine, inducing a decrease in the percentage of ciliated cells (Figure S7C), showing no effect on the expression of VDAC1-ΔC in hypoxia (Figure S7D), slightly increasing VDAC1 expression, not impacting on the metabolism of Cleavable#2 cells (Figure S7E,F), but increased both respirations and the glycolytic capacity were increased in #2 cells (Figure S7G,H).

We next questioned the mechanism by which VDAC links primary cilium and metabolism. We hypothesized that when VDAC1 is C-terminally truncated at asparagine 214, the putative double phosphorylation site at serine 215 [10] was lost leading to the release of tubulin (Figure S8A). Compacted tubulin was found around the nucleus in Vdac1^−/−^ and UnCleavable#2 cells, in comparison with Cleavable#2 cells in which tubulin appeared diffuse (Figure S8B). Co-immunoprecipitation studies were deployed to determine whether a physical interaction occurs between VDAC1 and tubulin βIII isoform. In Wt cells, endogenous VDAC1 co-immunoprecipitated with tubulin βIII in normoxia (Figure 4G). However, physical interactions seemed to be diminished in hypoxia suggesting the concept of a dissociation between VDAC1 and tubulin under hypoxic conditions. Then, we addressed the role of microtubule formation in the primary cilium. We confirmed the presence of microtubules close to mitochondrial membranes only in Vdac1^−/−^ and UnCleavable#2 cells (Figure S8C). A binding assay revealed a microtubule association with VDAC3 in Vdac1^−/−^ cells that was not impacted in hypoxia (Figure 4H and Figure S8D). However, monomers, dimers and tetramers of VDAC1 of Cleavable#2 cells in the pellet (P) were associated with microtubules in normoxia, whereas a clear dissociation was observed with the dimeric and tetrameric forms in hypoxia. In UnCleavable#2 cells, only the VDAC1 dimer in association with microtubules was observed in both normoxia and hypoxia suggesting no dissociation with the non-cleavable form of VDAC1.

These results reinforce the role of the VDAC1-tubulin/microtubule interaction in the regulation of mitochondrial metabolism and highlight a putative direct relationship between the cilium, VDAC1-ΔC and, for the first time, cell metabolism.

### 3.5. Direct Impact of VDAC1-ΔC on Cell Survival

**Figure 5:**
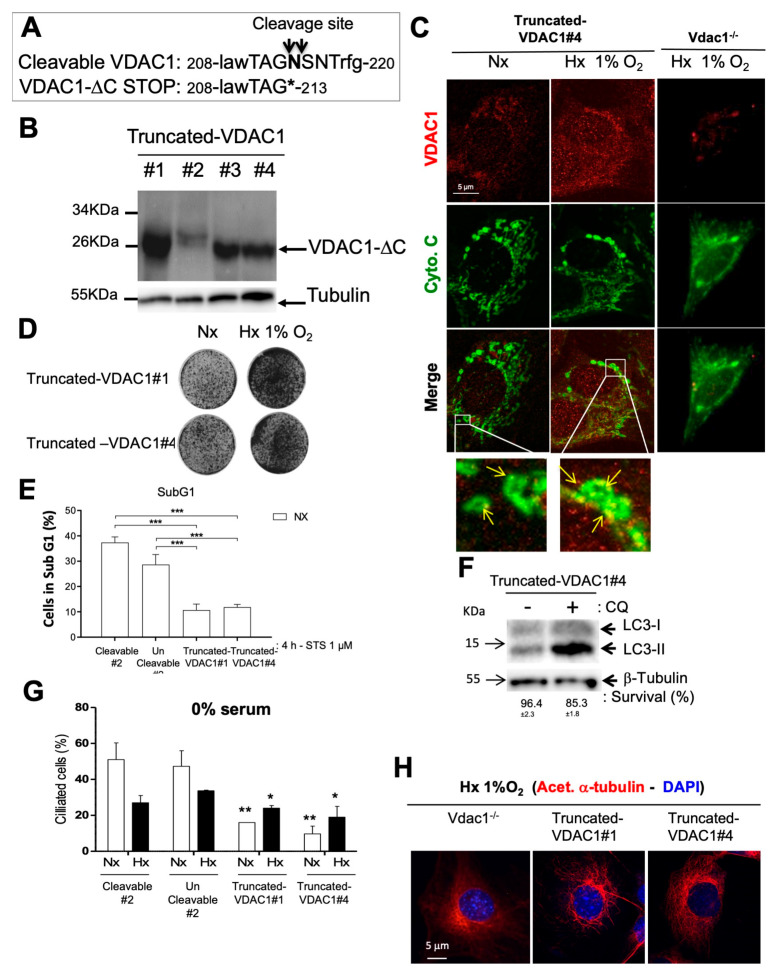
VDAC1-ΔC has a direct impact on cell death and primary cilium formation. (**A**) Schematic representation of mutations made at the cleavage site of VDAC1 to create a STOP Amber mutation. (**B**) Truncated-VDAC1#1/#2/#3/#4 cells were incubated in normoxia for 24 h and cell lysates were analyzed by immunoblotting for VDAC1. β-tubulin was used as a loading control. (**C**) Immunofluorescence to VDAC1 and cytochrome c (Cyto.C) in Truncated-VDAC1#4 cells in Nx and Hx 1% O2 and Vdac1^−/−^ cells in Hx 1% O_2_ for 72 h. (**D**) Clonogenic assay of Truncated-VDAC1#1 and #4 cells. Cell lines were seeded at the same density and incubated in Nx or Hx 1% O_2_ for 10 days. (**E**) Cleavable#2, Truncated-VDAC1#1/#4 cells were incubated in Nx or Hx 1% O_2_ for 72 h and challenged with staurosporin (STS) (1 µM) for 4 h. Cell mortality was measured using an ADAM cell counter. (**F**) Truncated-VADC1#4 cells were incubated in Nx in the absence (−) or presence (+) of chloroquine (CQ) and cell lysates were analyzed by immunoblotting for LC3-I/II. β-tubulin was used as a loading control. Cell viability was measured using an ADAM cell counter. (**G**) Quantitative analysis of the effect of Nx and Hx 1% O2 for 72 h on the ciliation percentage in the absence of serum (0% serum) in Cleavable#2, UnCleavable#2 and Truncated-VDAC1#1/#4 cells assessed by confocal fluorescence microscopy (*n* = 100–300 cells). (**H**) Immunofluorescence to acetylated α-tubulin (acet. α-tubulin in red) and DAPI (in blue) in Truncated-VDAC1#1 and Truncated-VDAC1#4 cells in Hx 1% O_2_ for 72 h. * indicates *p* < 0.05 and ** indicates *p* < 0.005. The uncropped Western Blot figures in Figure S10.

In order to obtain a cleaved form of VDAC1 in normoxia (i.e., a truncated isoform terminated at codon #214), a STOP Amber mutation was introduced in the cleavage site of human VDAC1 (Figure 5A). Plasmids expressing Truncated-VDAC1, which is similar to VDAC1-ΔC, were stably transfected into Vdac1^−/−^ Ras MEFs. Four Ras MEFs clones expressing Truncated-VDAC1 (Truncated-VDAC1#1/2/3/4) were obtained and tested in normoxia for protein expression levels (Figure 5B). Only two of these clones were used and referred to as Truncated-VDAC1#1 and #4 cells. Truncated-VDAC1#1/#4 cells exposed to normoxia and hypoxia showed VDAC1 staining, enlarged mitochondria and enhanced growth in hypoxia compared to normoxia (Figure 5C,D and Figure S9A). Truncated-VDAC1#1/#4 cells, incubated with staurosporine (STS), exhibited little apoptosis in normoxia after 72 h compared to Wt or UnCleavable#2 (Figure 5E). Moreover, autophagy flux, using LC3-II expression, was observed in Truncated-VDAC1#4 cells in normoxia and cells were protected from cell death when CQ was added (Figure 5F). To further characterize the role of VDAC1-ΔC in ciliogenesis, primary cilia were counted in Truncated-VDAC1#1/#4 cells in normoxia and hypoxia (Figure 5G). Truncated-VDAC1#1/#4 cells showed less primary cilia than Cleavable#2 or UnCleavable#2 cells in both normoxia or hypoxia, in conditions where cell cycle was controlled and similar (0% serum—Figure 5G). Tubulin network was very spread out in the cytoplasm (Figure 5H). Together, these results directly proved for the first time that VDAC1-ΔC, per se, confers a survival advantage to Ras MEFs in addition to negatively regulate ciliogenesis.

### 3.6. Direct Impact of VDAC1-ΔC on Cell Metabolism

**Figure 6:**
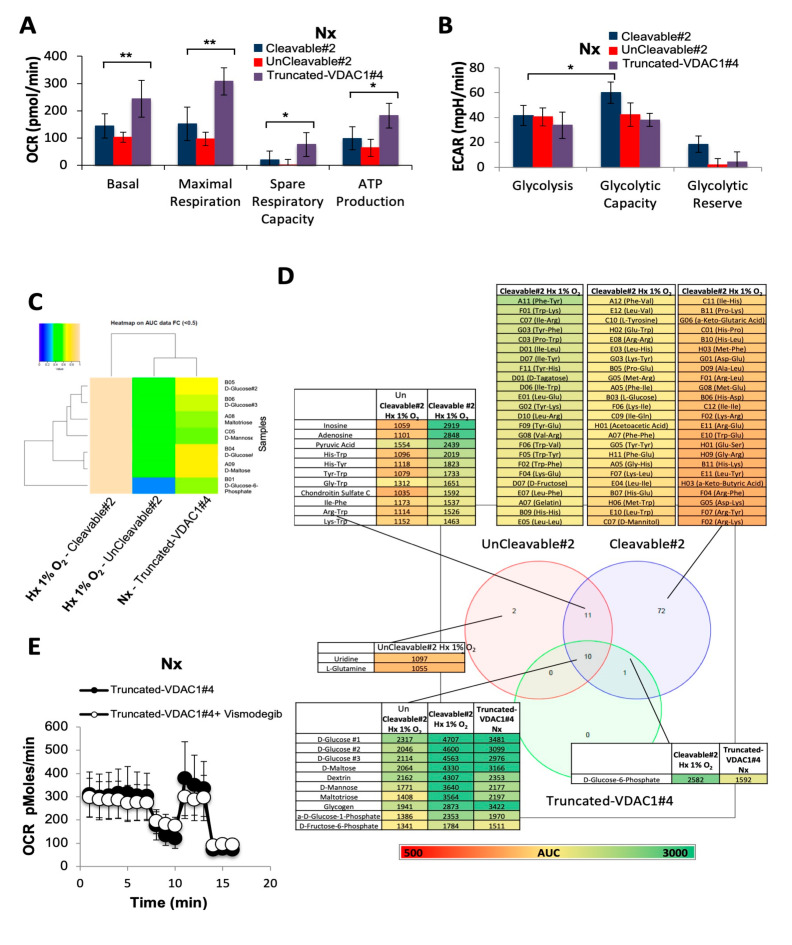

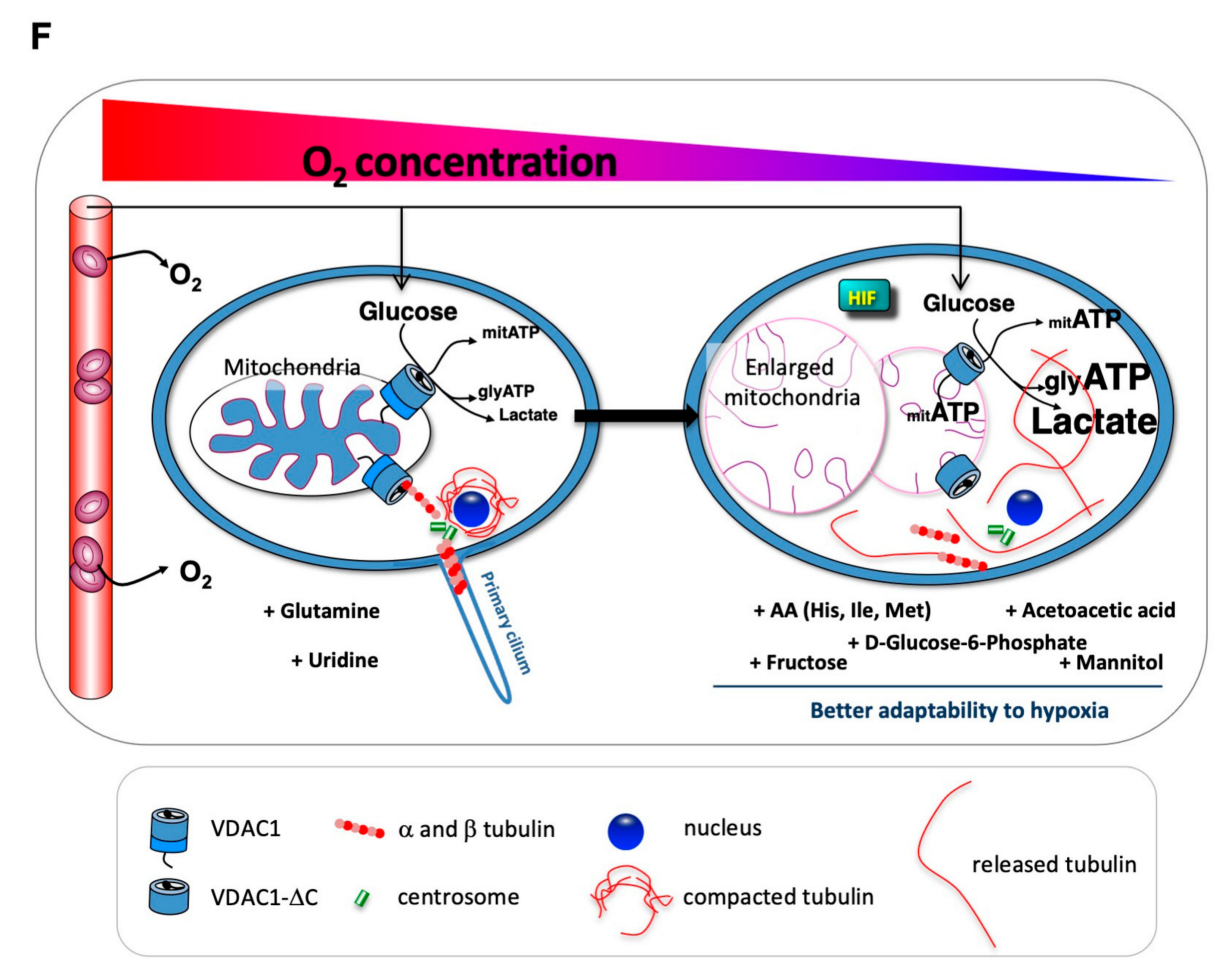
VDAC1-ΔC has a direct impact on metabolism in normoxia. (**A**) Respiratory control of Cleavable#2, UnCleavable#2 and Truncated-VDAC1#4. OCR was measured in real time with the XF24 analyzer in Nx. The mean ± SEM is representative of at least three independent experiments carried out in quadruplicate. (**B**) ECAR in Nx of Cleavable#2, UnCleavable#2 and Truncated-VDAC1#4 was evaluated with with the XF24 analyzer. The mean ± SEM is representative of at least three independent experiments carried out in quadruplicate. (**C**) Heatmap showing the 7 substrates that were communly metabolized by Cleavable#2, UnCleavable#2, and Truncated-VDAC1#4 in normoxia. The color key scale for each substrate is based on dye reduction quantified by Omnilog units. A yellow color indicates strong positive substrate metabolization, a green color moderate metabolization and a blue color indicates no substrate metabolization. Data area under the curve (AUC) < 500 have been excluded. (**D**) Venn diagram comparing metabolic phenotyping of Cleavable#2 in hypoxia, UnCleavable#2 in hypoxia and Truncated-VDAC1#4 in normoxia. Data AUC > 500. (**E**) Respiratory control of Truncated-VDAC1#4 in the presence of Vismodegib (50 µM). OCR was measured in real time with the XF24 analyzer normoxia (Nx). Cells were deprived of glucose for 1 h, then glucose (G), oligomycin (O), FCCP (F), and Rotenone + Antimycin A (R/A) were injected at the indicated times. (**F**) Graphical model. * indicates *p* < 0.05 and ** indicates *p* < 0.005.

Quantification of mitochondrial respiration (OCR) and glycolytic capacity (ECAR) in Truncated-VDAC1#4 cells compared to Cleavable#2 and UnCleavable#2 cells clearly showed a higher basal respiration and maximal respiration in normoxia (Figure 6A). However, Truncated-VDAC1#4 cells showed similar basal level of glycolysis compared to Cleavable#2 and UnCleavable#2 in normoxia, and maximal glycolytic capacity and glycolytic reserve similar to UnCleavable#2 (Figure 6B). Using the OmniLog Phenotype MicroArray to probe metabolism, we demonstrated that Truncated-VDAC1#4 cells had a normoxic metabolism closer to the hypoxic metabolism of Cleavable#2 compared to UnCleavable#1/#2 cells (Figure 6C,D). Finally, inhibition of ciliogenesis with vismodegib did not affect either the respiration (Figure 6E) or the glycolysis (data not shown).

These genetic experiments provide proof of concept that the presence of VDAC1-ΔC in the absence of VDAC1 can confer a higher metabolism and thus a better adaptability.

## 4. Discussion

Our data describe first a new mechanism for the control of metabolism that is driven by VDAC1-ΔC, the form of VDAC1 that is produced in hypoxia in many cancer cells or patients as previously described [7,8,9,13] and second, a link between VDAC1, tubulin, microtubules, and primary cilium.

The role of VDAC in metabolic homeostasis has been studied extensively [4,20,21,22,23,24,25,26,27,28,29]. However, an extremely important role as a metabolic checkpoint has been attributed to VDAC: controlling the Warburg effect, which enhances glycolysis and represses mitochondrial metabolism in cancer [10,11,12,30,31]. By regulating the transport functionality via pore opening of VDAC, free tubulin, specifically tubulin βII and βIII isoforms, operates as a “master key” controlling mitochondrial metabolism [32]. For many years, we and others have been investigating the role of VDAC1-ΔC in hypoxia [7,8,9] and under iron deprivation conditions [33]. Our present study describes, for the first time, a mechanism in which hypoxic cancer cells can access new energy sources to promote cell survival in response to a changing microenvironment. However, the cells not only produce more energy, but unexpectedly, they develop metabolic flexibility driven by VDAC1-ΔC. By using D-glucose, glucose, maltose, maltotriose, and D-glucose-6-phosphate, five important carbohydrates involved in glucose metabolism, hypoxic cancer cells expressing VDAC1-ΔC, with or without VDAC1, could exhibit faster and sustained growth for a long period of energy expenditure and stress. Whether cancer cells presenting VDAC1-ΔC have an increased demand for sugars is not yet known, but we have clearly demonstrated that they can use a wider range of sugars that can be used in the glycolytic pathway. VDAC1, and more specifically VDAC1-ΔC, is therefore at the center of the regulation and control of the Warburg effect, the most prominent hallmark of cancer metabolism.

Interestingly, our study of association/dissociation of VDAC1/tubulin reveals a novel function in controlling the biogenesis of the primary cilium. In line with previous works [10,11,30,31,34], we characterized the differential role of phosphorylated sites involved in the interaction between VDAC1 and tubulin. Upon cleavage of VDAC1 in hypoxia by LGMN at serine 214, phosphorylation at position 211 is preserved, but the 215 phosphorylation pattern is lost, hampering the binding between tubulin and the dimeric/tetrameric forms of VDAC1 in hypoxia. This result indicates that phosphorylation at position 215 is extremely important for this interaction. Moreover, we discovered that oligomerization of VDAC1 is crucial in this process. The importance of VDAC1 oligomers has previously been described in apoptosis, where they form a pore large enough to mediate the passage of cytochrome c [29,35]. However, the importance of the VDAC1 oligomerization on its interaction with tubulin and its participation in the biogenesis of the primary cilium remained unknown. CTTs of tubulin could sterically and electrostatically fit in the positively-charged VDAC pore, as proposed by Rostovtseva et al. [10], and in retinal cells, could involve Mps1, a protein kinase that plays a role in centriole assembly to negatively regulate ciliogenesis [15]. However, in cancer cells, our preliminary results showed that VDAC1 is in close proximity to the centrosome (data not shown) suggesting that VDAC1 could participate in microtubule nucleation, a mechanism that we are exploring further.

Finally, there was still limited information about the role of the primary cilium in metabolism in general and in cancer metabolism in particular [36]. Lee et al. [37] recently demonstrated that the loss of function of intraflagellar transport protein 88 homolog (IFT88) combined with the loss of primary cilium resulted in mitochondrial dysfunction in favor of glycolytic metabolism and lipid biosynthesis. By decreasing the number of ciliated cells using colchicine, a microtubule destabilizer, or vismodegib, a Hedgehog signaling pathway inhibitor, both of which are used as anti-cancer treatments, glycolysis and respiration are clearly favored in the absence of VDAC1-ΔC. On the other hand, by using taxol, a mitotic inhibitor preventing cancer cells from dividing, the proportion of ciliated VDAC1-ΔC cell was increased, resulting in a decrease in both glycolysis and respiration. One could speculate that the primary cilium is indirectly regulating metabolism via the VDAC1 pore, possibly mediating some of the side effects observed during treatments in patients.

## 5. Conclusions

This study highlights two original major characteristics of VDAC1-ΔC, one associated with the control of metabolism flexibility and the other with the control of the primary cilium that was unexpected. As part of an integrated model favoring a better adaptability to hypoxia (Figure 6F), VDAC1-ΔC appears as a new biomarker with a potential in unraveling basic mechanisms of cancer and ciliopathy development.

## Supplementary Materials

The following are available online at https://www.mdpi.com/2072-6694/12/11/3484/s1, Figure S1: Vdac1 null (*Vdac1*^−/−^) cells expressing wild-type VDAC1 (Cleavable#1/#2) are more resistant to cell death than Vdac1 null cells expressing VDAC1 mutated at the VDAC-DC cleavage site (*N*-Cleavable#1/2) in 1% O_2_ hypoxia (Hx 1% O_2_), Figure S2: Metabolic characteristics of Vdac1 null (*Vdac1*^−/−^) cells expressing wild-type VDAC1 (Cleavable#1/#2) and Vdac1 null cells expressing VDAC1 mutated at the VDAC-DC cleavage site (*N*-Cleavable#1/#2) of RASV12-transformed mouse embryonic fibroblasts (Ras MEFs) in long-term hypoxia (15 days), Figure S3: Metabolic characteristics of Vdac1 null (*Vdac1*^−/−^) cells expressing wild-type VDAC1 (Cleavable#1/#2) and Vdac1 null cells expressing VDAC1 mutated at the VDAC-DC cleavage site (*N*-Cleavable#1/#2) of RASV12-transformed mouse embryonic fibroblasts (Ras MEFs) in normoxia (Nx), Figure S4: Lactate and ATP productions of Cleavable#1/#2 compared to *N*-Cleavable#1/#2, Figure S5: Additional informations on metabolic phenotyping of Cleavable#2 compared to *N*-Cleavable#2, Figure S6: VDAC3 maintains biogenesis of the primary cilium in *Vdac1*^−/−^ Ras MEF, Figure S7: Vismodegib, a Hedgehog signaling pathway inhibitor, decreased the percentage of ciliation and modified metabolism, which depended on the cleaved form of VDAC1, Figure S8: The hypoxic VDAC-ΔC form loses interaction with tubulin, Figure S9: Characteristics of Truncated-VDAC1#1,Figure S10. Uncropped Western Blot Figures.
