# Supplementary material for: Evidences of a Direct Relationship between Cellular Fuel Supply and Ciliogenesis Regulated by Hypoxic VDAC1-ΔC
Source: Cancers (Basel). 2020 Nov 23;12(11):3484. doi: 10.3390/cancers12113484 (PMC7700438; doi:10.3390/cancers12113484)
Supplement: Supplementary file 1 [file cancers-12-03484-s001.pdf]

Supplementary Materials: Evidences of a Direct Relationship between Cellular Fuel Supply and Ciliogenesis Regulated by Hypoxic VDAC1-ΔC

Monique Meyenberg Cunha-de Padua, Lucilla Fabbri, Maeva Dufies, Sandra Lacas-Gervais, Julie Contenti, Charles Voyton, Sofia Fazio, Marie Irondelle, Baharia Mograbi, Matthieu Rouleau, Nirvana Sadaghianloo, Amandine Rovini, Catherine Brenner, William J. Craigen, Jérôme Bourgeais, Olivier Hérault, Frédéric Bost and Nathalie M. Mazure

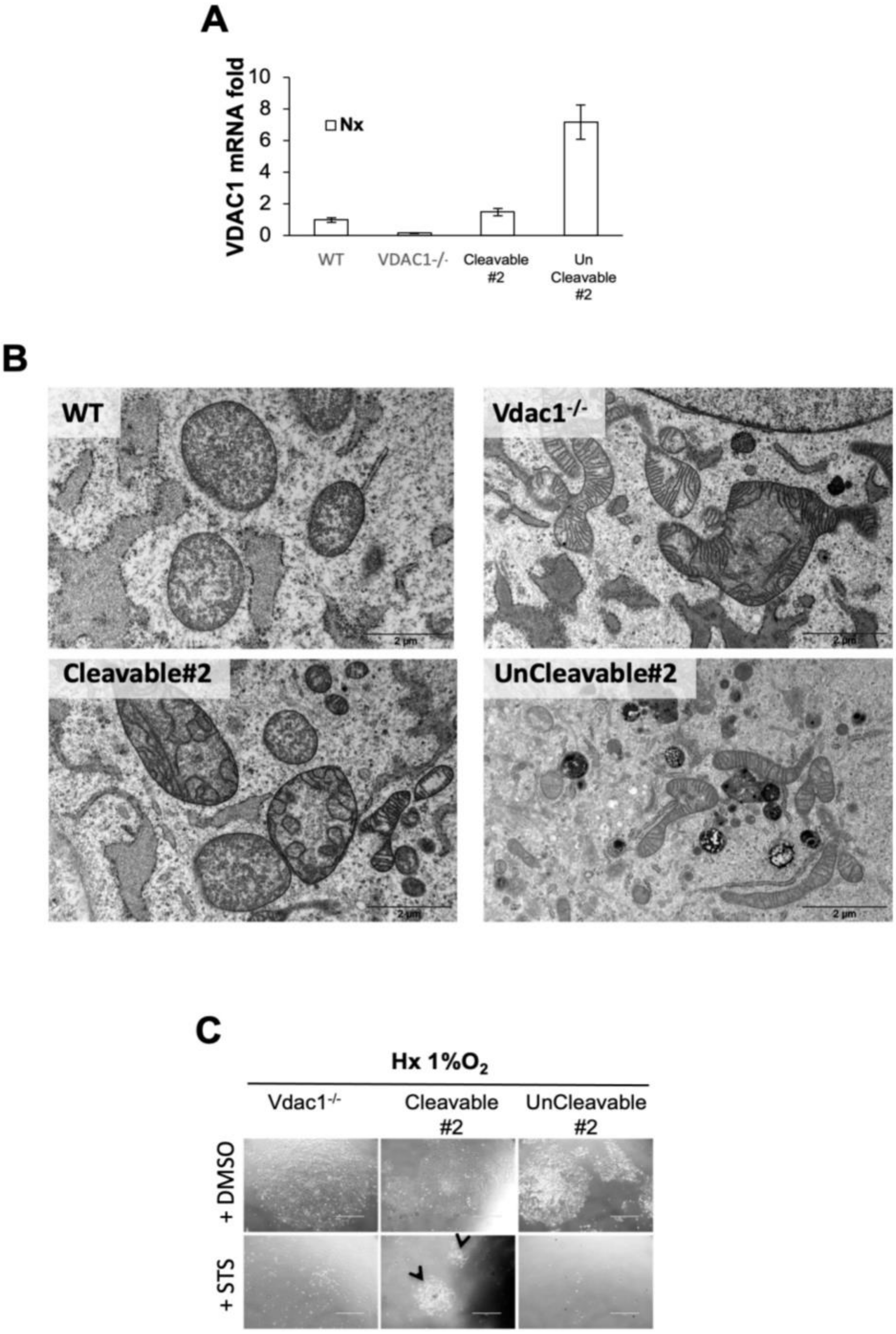

Figure S1. Vdac1 null (*Vdac1*$^{-/-}$) cells expressing wild-type VDAC1 (Cleavable#1/#2) are more resistant to cell death than Vdac1 null cells expressing VDAC1 mutated at the VDAC-DC cleavage site (*N*-Cleavable#1/2) in 1% O_2 hypoxia (Hx 1% O_2). (**A**) Histograms represent the expression of VDAC1 mRNA in Wt, *Vdac1*$^{-/-}$, Cleavable#2 and *N*-Cleavable #2 cells in Nx for 72 h. The mean±SEM

is representative of three independent experiments. (**B**) *Vdac1*$^{-/-}$, Cleavable #2 and *N*-Cleavable #2 cells were incubated in Hx 1% O_2 for 72 h and cell lysates were analyzed by immunoblotting for Flag (mouse monoclonal antibody). β-tubulin was used as a loading control. (**C**) Representative electron micrographs of mitochondria of Wt, *Vdac1*$^{-/-}$, Cleavable #2 and *N*-Cleavable #2 cells incubated in Hx 1% O_2 for 72 h. (**D**) Representative photomicrographs of clones of *Vdac1* Cleavable #1/#2 and *N*-Cleavable #1/#2 cells incubated in Hx 1% O_2 for 8 days in the absence (+DMSO) or presence of staurosporine (+STS) for the last 5 days.

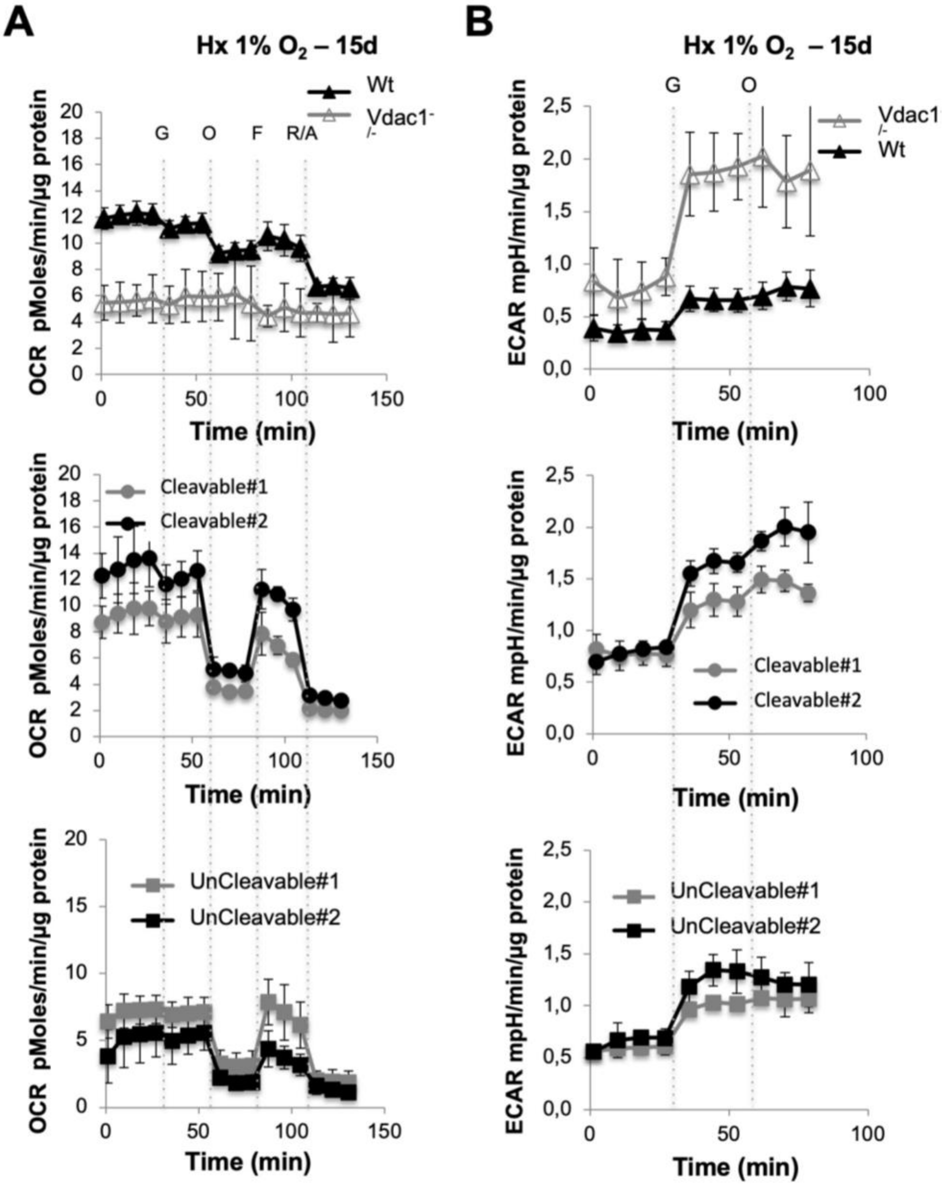

Figure S2. Metabolic characteristics of Vdac1 null (*Vdac1*$^{-/-}$) cells expressing wild-type VDAC1 (Cleavable#1/#2) and Vdac1 null cells expressing VDAC1 mutated at the VDAC-DC cleavage site (*N*-Cleavable#1/#2) of RASV12-transformed mouse embryonic fibroblasts (Ras MEFs) in long-term hypoxia (15 days). (**A**) Respiratory control of Wt and *Vdac1*$^{-/-}$ cells (top panel), Cleavable#1/#2 (middle panel) and *N*-Cleavable#1/#2 (bottom panel). OCR was measured in real time with the XF24 analyzer in Hx 1% O_2. Cells were deprived of glucose for 1 h, then glucose (G), oligomycin (O), FCCP (F) and Rotenone + Antimycin A (R/A) were injected at the indicated times. The mean±SEM is representative of at least three independent experiments carried out in quadruplicate. (**B**) ECAR in Hx 1% O_2 of Wt and *Vdac1*$^{-/-}$ cells (top panel), Cleavable#1/#2 (middle panel) and *N*-Cleavable#1/#2 (bottom panel) was evaluated with the XF24 analyzer. Cells were deprived of glucose for 1 h, then glucose (G) and oligomycin (O) were injected at the indicated times. The mean±SEM is representative of at least three independent experiments carried out in quadruplicate.

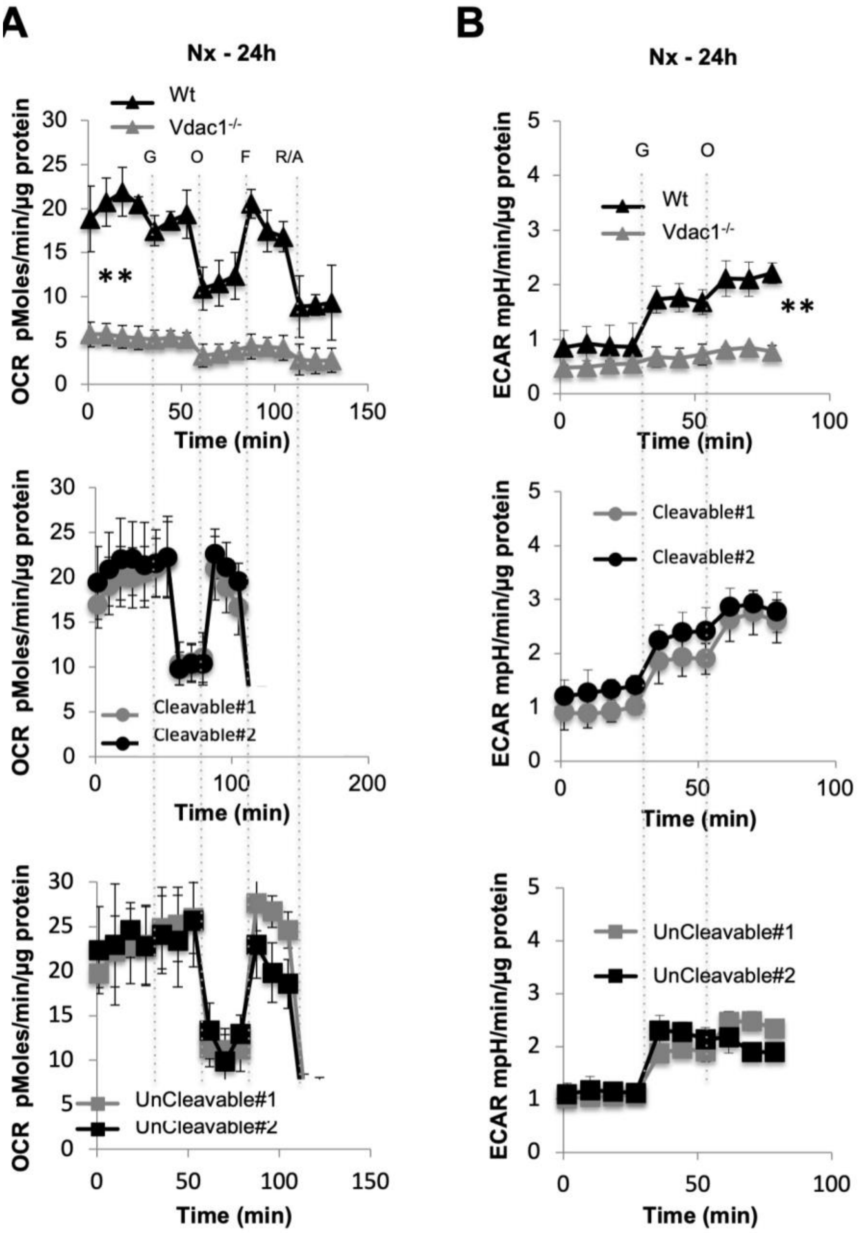

Figure S3. Metabolic characteristics of Vdac1 null (*Vdac1*$^{-/-}$) cells expressing wild-type VDAC1 (Cleavable#1/#2) and Vdac1 null cells expressing VDAC1 mutated at the VDAC-DC cleavage site (*N*-Cleavable#1/#2) of RASV12-transformed mouse embryonic fibroblasts (Ras MEFs) in normoxia (Nx). (**A**) Respiratory control of Wt and *Vdac1*$^{-/-}$ cells (top panel), Cleavable #1/#2 (middle panel) and *N*-Cleavable#1/#2 (bottom panel). Oxygen Consumption Rate (OCR) was measured in real time with the XF24 analyzer in Hx. Cells were deprived of glucose for 1 h, then glucose (G), oligomycin (O), FCCP (F) and Rotenone + Antimycin A (R/A) were injected at the indicated times. The mean±SEM is representative of at least three independent experiments carried out in quadruplicate. (**B**) The ExtraCellular Acidification Rate (ECAR) in Hx of Wt and *Vdac1*$^{-/-}$ cells (top panel), Cleavable#1/#2 (middle panel) and *N*-Cleavable#1/#2 (bottom panel) was evaluated with the XF24 analyzer. Cells were deprived of glucose for 1 h, then glucose (G) and oligomycin (O) were injected at the indicated times. The mean±SEM is representative of at least three independent experiments carried out in quadruplicate. ** A ** $p < 0.005$ show significant differences.

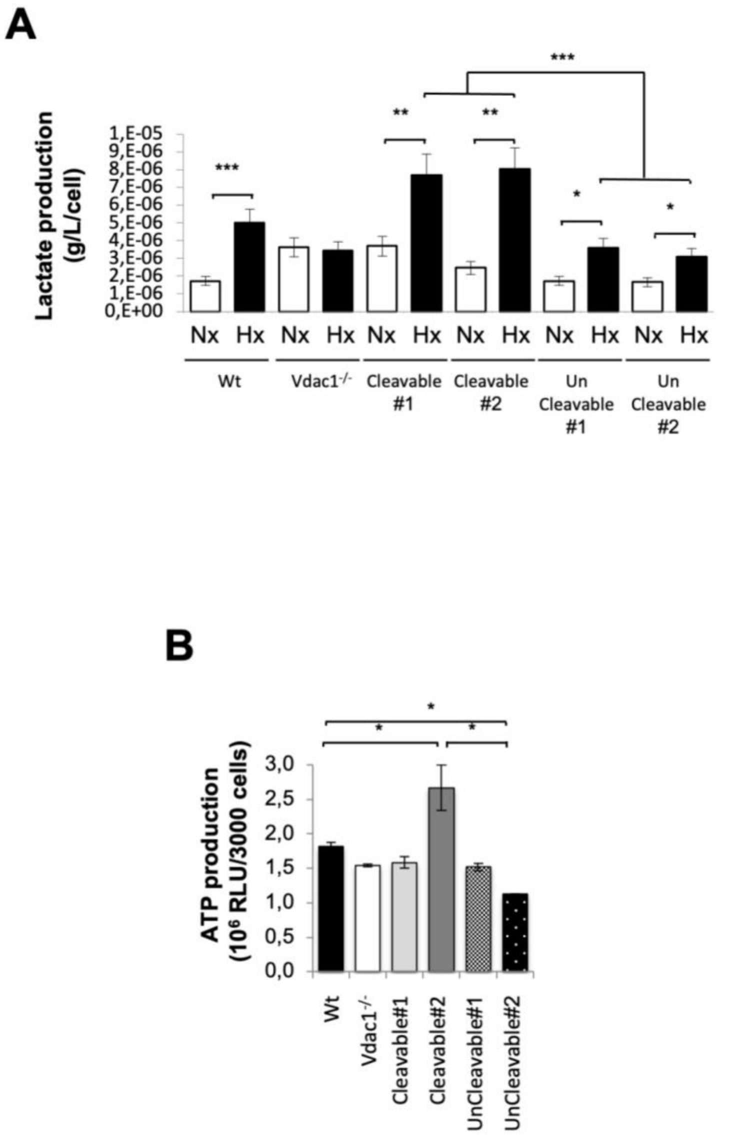

Figure S4. Lactate and ATP productions of Cleavable#1/#2 compared to *N*-Cleavable#1/#2. (**A**) After 72 h of culture in Nx or Hx 1% O_2, cells were lysed in Assay Buffer with sonication. The amount of lactate was quantified in cell extracts. The mean ± SEM is representative of three independent experiments carried out in duplicate. (**B**) ATP production in Wt, *Vdac1*$^{-/-}$, Cleavable#1/#2 and *N*-Cleavable#1/#2 cells in Hx 1% O_2 for 72 h. The mean±SEM is representative of three independent experiments carried out in duplicate. A * $p < 0.05$, ** $p < 0.005$ and *** $p < 0.0005$ show significant differences.

A

Dimension 1

		correlation	p.value
C05	(His-Val)	0.9996825	0.00031755
B11	(His-Lys)	0.9992251	0.00077487
C03	(His-Trp)	0.9986331	0.00136687
C07	(Ile-Arg)	0.9977579	0.00224213
C11	(Ile-His)	0.9938191	0.0061809
E07	(Leu-Phe)	0.9922729	0.00772707
F08	(Lys-Lys)	0.9922596	0.00774043
E12	(Leu-Val)	0.9908759	0.0091241
D07	(Ile-Tyr)	0.9879925	0.01200752
D01	(Ile-Leu)	0.9863241	0.01367593
H03	(Met-Phe)	0.9839526	0.01604744
B07	(His-Glu)	0.9831862	0.01681376
G08	(Met-Glu)	0.9828928	0.01710723
F10	(Lys-Phe)	0.982223	0.01777704
D06	(Ile-Trp)	0.9758916	0.02410836
G10	(Met-His)	0.9750914	0.02490864
E01	(Leu-Glu)	0.9727516	0.02724837
B10	(His-Leu)	0.9701969	0.02980311
E08	(Leu-Pro)	0.9689165	0.03108346
C07	(D-Mannitol)	0.968889	0.03111097
B01	(Gly-Thr)	0.9677772	0.03222283
D01	(D-Tagatose)	0.9670684	0.0329316
G01	(Lys-Thr)	0.9659698	0.03403021
E11	(Leu-Tyr)	0.9657546	0.03424538
E05	(Leu-Leu)	0.9655441	0.03445592
C12	(Ile-Ile)	0.9654733	0.03452673
H01	(Glu-Ser)	0.9653227	0.03467727
G11	(Met-Ile)	0.9642976	0.03570242
F04	(Lys-Glu)	0.9633909	0.03660911
H07	(Met-Tyr)	0.9625359	0.03746407
H06	(Met-Trp)	0.9620646	0.0379354
A06	(Glycogen)	0.9618173	0.03818274
C01	(His-Pro)	0.9612112	0.03878883
D07	(D-Fructose)	0.959599	0.04040103
A10	(Gly-Phe)	0.9569827	0.04301729
F07	(Lys-Leu)	0.9553322	0.04466776
D08	(Ile-Val)	0.95381	0.04618996
F06	(Lys-Ile)	0.9538008	0.04619918
A07	(Gelatin)	0.9537382	0.04626182
H11	(Phe-Glu)	0.9533744	0.04662563
E08	(Arg-Arg)	0.952536	0.04746397
H03	(a-Keto-Butyric Acid)	0.9507025	0.04929751
E06	(Leu-Met)	0.9502669	0.04973305
C10	(L-Tyrosine)	0.950087	0.04991295

Dimension 2

		correlation	p.value
C02	(D-Glucuronic Acid)	0.9884732	0.01152685
B03	(Gly-Tyr)	0.9783725	0.02162753
E03	(D-Galactose)	0.9772213	0.02277868
D02	(Ile-Met)	0.962812	0.03718803
D04	(L-Fucose)	0.9605089	0.03949108
B10	(D-Salicin)	0.9569541	0.04304589
F10	(Asn-Val)	0.9549543	0.04504566
F05	(Xylitol)	0.9547163	0.04528373
C05	(D-Mannose)	-0.9650389	0.03496112

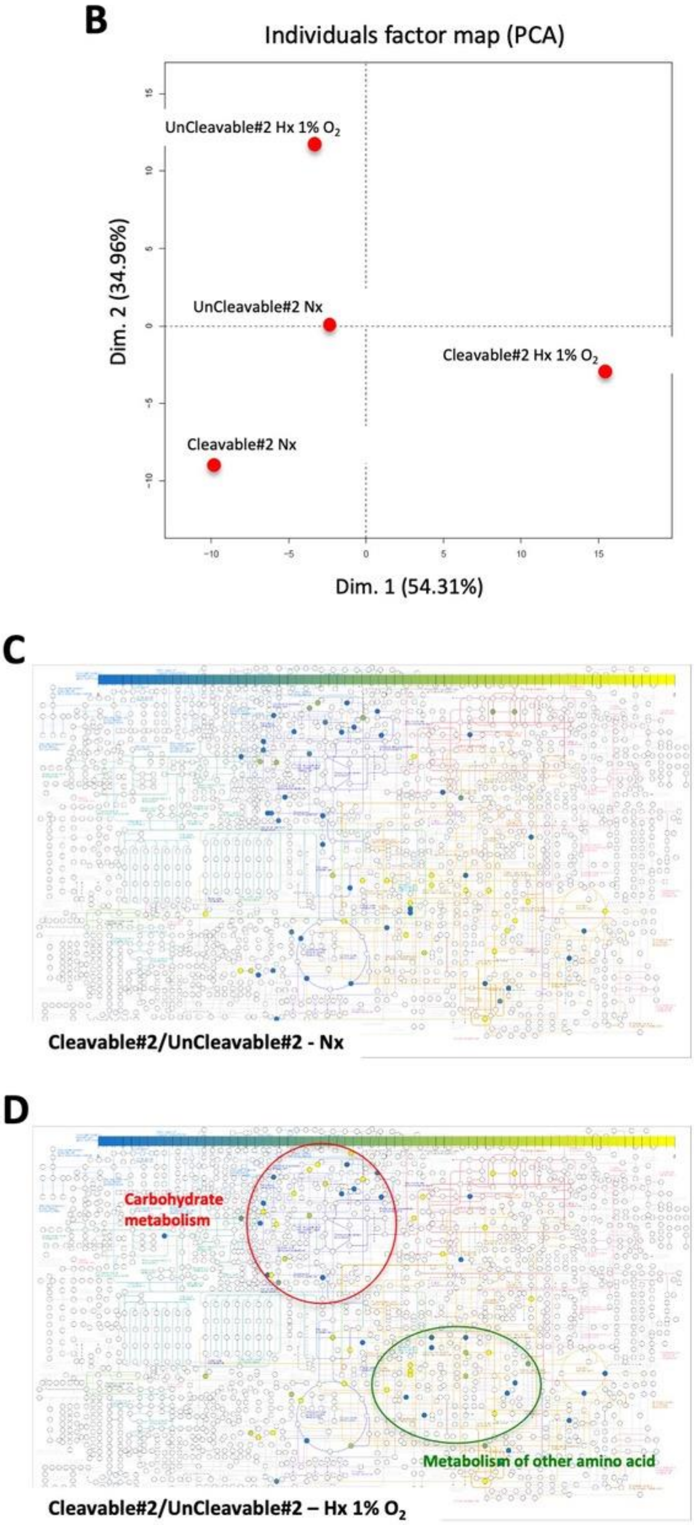

Figure S5. Additional informations on metabolic phenotyping of Cleavable#2 compared to *N*-Cleavable#2. (**A**) List of the different substrates allowing the map representation (PCA) representing 2 dimensions (dim. 1 and 2). (**B**) Map representation (PCA) representing 2 dimensions (dim. 1 and 2). PCA was based on the analyzed metabolites with an AUC > 500. (**C**) and (**D**) Substrate mapping, via KEGG metabolic pathway, used by Cleavable#2 compared to *N*-Cleavable#2 in normoxia (Nx—(**C**)) and hypoxia (Hx 1% O_2 —(**D**)).

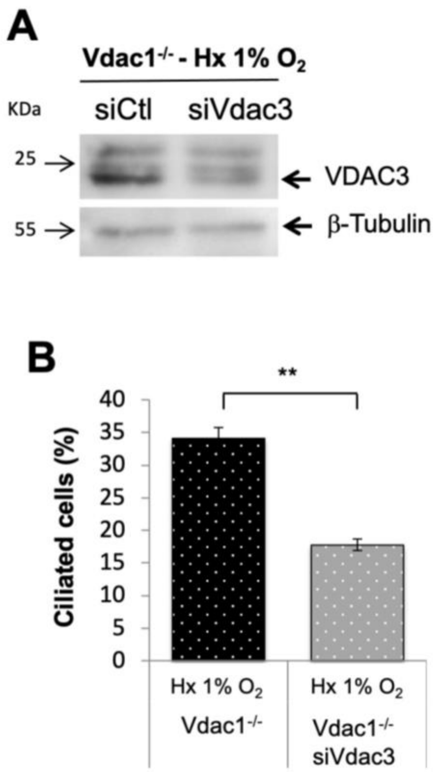

Figure S6. VDAC3 maintains biogenesis of the primary cilium in *Vdac1*$^{-/-}$ Ras MEF. (**A**) *Vdac1*$^{-/-}$ cells were incubated in Hx 1% O_2 for 72 h and cell lysates were analyzed by immunoblotting for VDAC3. β-tubulin was used as a loading control. (**B**) Quantitative analysis of the effect of siRNA to VDAC3 in Hx 1% O_2 for 72 h on the ciliation percentage in the presence of 20% serum in *Vdac1*$^{-/-}$ cells as assessed by confocal fluorescence microscopy (n = 100–300 cells). A ** $p < 0.005$ shows significant differences.

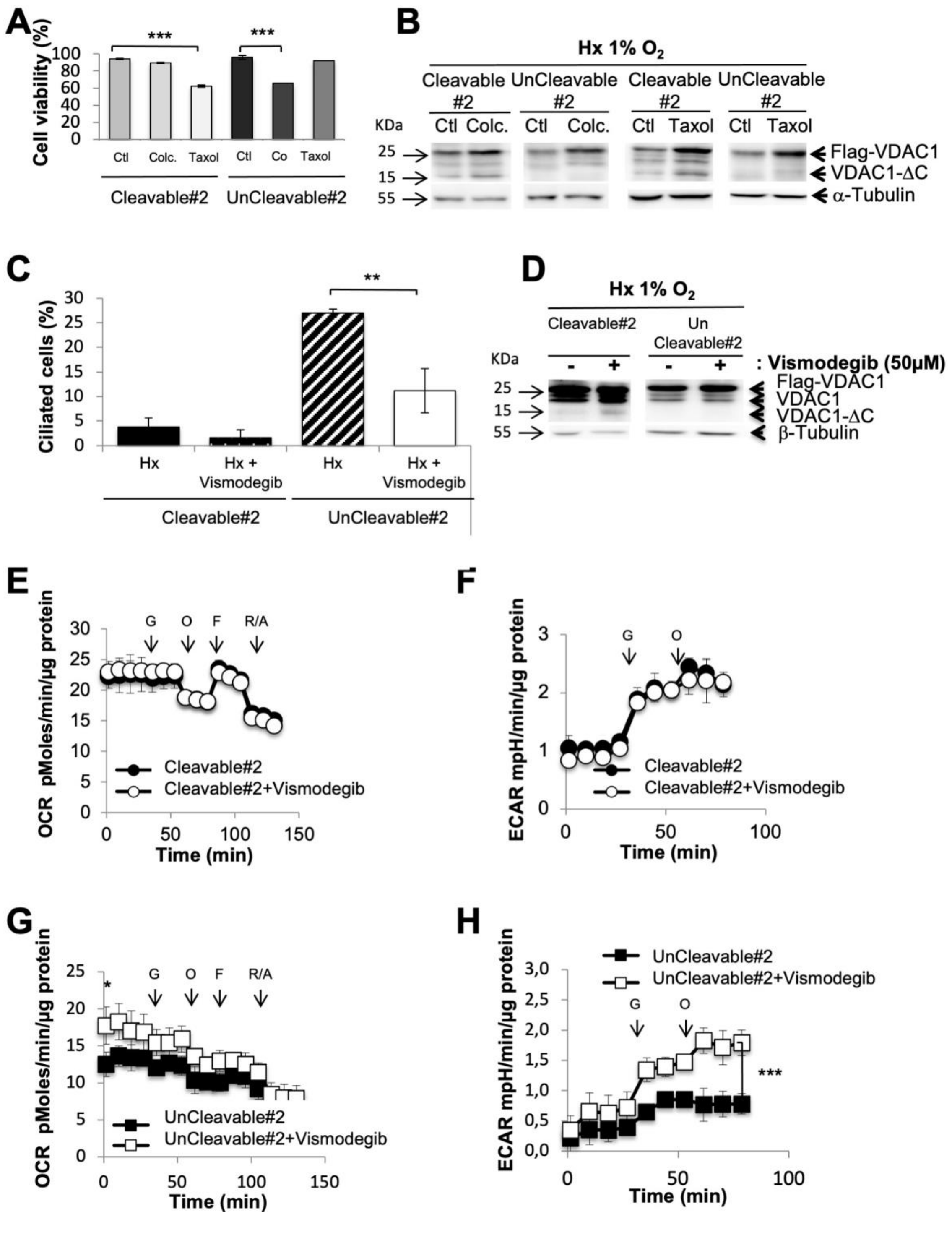

Figure S7. Vismodegib, a Hedgehog signaling pathway inhibitor, decreased the percentage of ciliation and modified metabolism, which depended on the cleaved form of VDAC1. (**A**) Cleavable#2 and *N*-Cleavable#2 cells were incubated in the absence or presence of colchicine (Colc.–1 μM) or taxol (1 μM) in Hx 1% O_2 for 72 h and cell viability was measured using an ADAM cell counter. (**B**) Cleavable#2 and *N*-Cleavable #2 cells were incubated in the absence or presence of colchicine (Colc.–1 μM) or taxol (1 μM) in Hx 1% O_2 for 72 h and cell lysates were analyzed by immunoblotting for VDAC1. β-tubulin was used as a loading control. (**C**) Quantitative analysis of the effect of the absence or presence of Vismodegib (50 μM) for 72 h on the percentage of ciliation in Cleavable#1/#2 and *N*-Cleavable#1/#2 cells assessed by confocal fluorescence microscopy (n = 100–300 cells). (**D**) Cleavable#1/#2 and *N*-Cleavable #1/#2 cells were incubated in Hx 1% O_2 in the absence or presence of Vismodegib (50 μM) for 72 h and cell lysates were analyzed by immunoblotting for VDAC1. β-tubulin was used as a loading control. (**E**) and (**F**) Respiratory control of Cleavable#2 (**E**)

and *N*-Cleavable#2 cells (**F**) in the presence of Vismodegib (50 μM). OCR was measured in real time with the XF24 analyzer in Hx 1% O_2. Cells were deprived of glucose for 1 h, then glucose (G), oligomycin (O), FCCP (F) and Rotenone + Antimycin A (R/A) were injected at the indicated times. (**G**) and (**H**) ECAR in Hx of Cleavable#2 (**G**) and *N*-Cleavable#2 (**H**) in the presence of Vismodegib (50 μM) was evaluated with the XF24 analyzer. Cells were deprived of glucose for 1 h, then glucose (G) and oligomycin (O) were injected at the indicated times. A * $p < 0.05$, ** $p < 0.005$ and *** $p < 0.0005$ show significant differences.

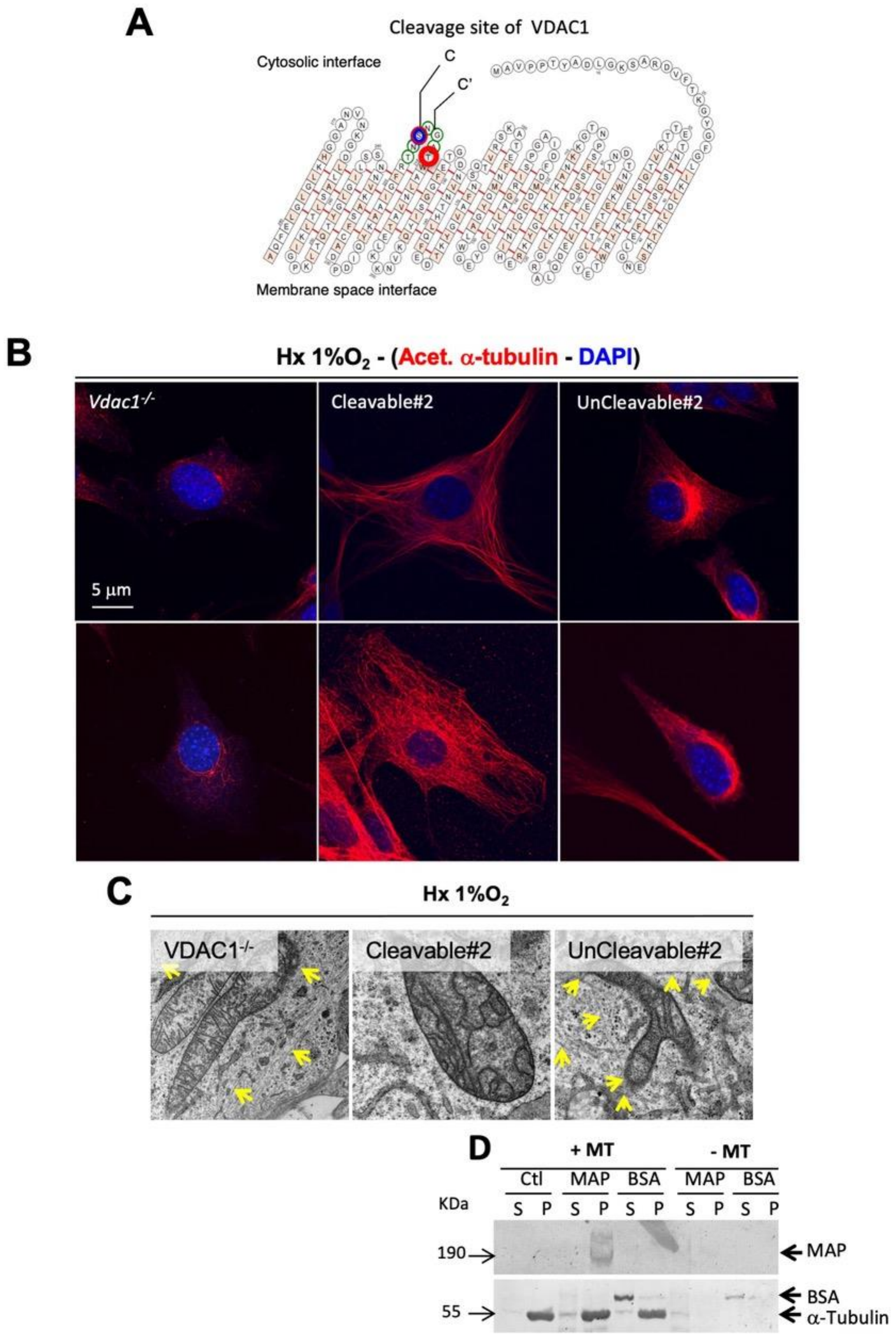

Figure S8. The hypoxic VDAC-ΔC form loses interaction with tubulin. (**A**) Structure of VDAC1 showing the major cleavage site of VDAC1 C-terminal to asparagine 214 (C) with minor cleavage at glycine 213 (C′) and the major VDAC phosphorylation sites. Phosphorylation sites of PKA (circled in blue) and GSK3b (circled in red) are located on loops L5 and L7, facing the cytosolic side and flanking the cleavage site of VDAC1. (**B**) Immunofluorescence to acetylated α-tubulin (acet. α-tubulin in red) and DAPI (in blue) in *Vdac1*$^{-/-}$, Cleavable#2 and *N*-Cleavable#2 cells in Hx for 72 h. (**C**) Representative electron micrographs of mitochondria and microtubules of *Vdac1*$^{-/-}$, Cleavable#2 and *N*-Cleavable#2 cells incubated in hypoxia (Hx 1% O_2) for 72 h. Yellow arrows show microtubules close to mitochondria. (**D**) Controls of the pelleting assay for microtubule-associated proteins. Wt cells were incubated in the absence (−MT) or presence of Taxol-stabilized microtubules (+MT) and then pelleted through a 40% glycerol cushion. MAP, BSA and a-tubulin in supernatant (S) and pellet (P) were analyzed by immunoblotting.

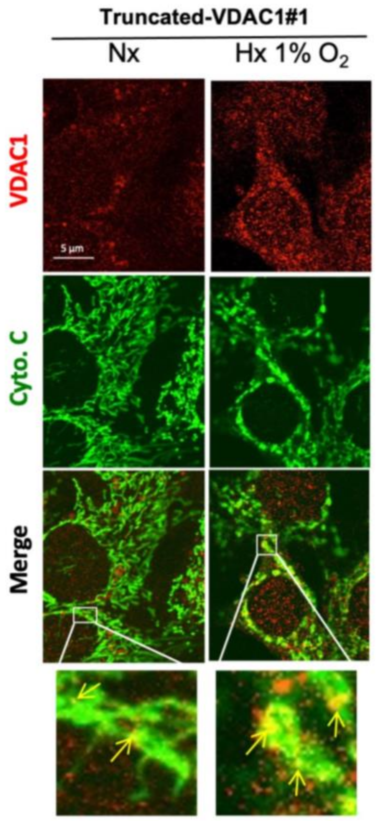

Figure S9. Characteristics of Truncated-VDAC1#1. Immunofluorescence to VDAC1 and cytochrome c (Cyto.C) in Truncated-VDAC1#1 cells in Nx and Hx 1% O_2 for 72 h.

Figure S10. Uncropped Western Blot Figures.

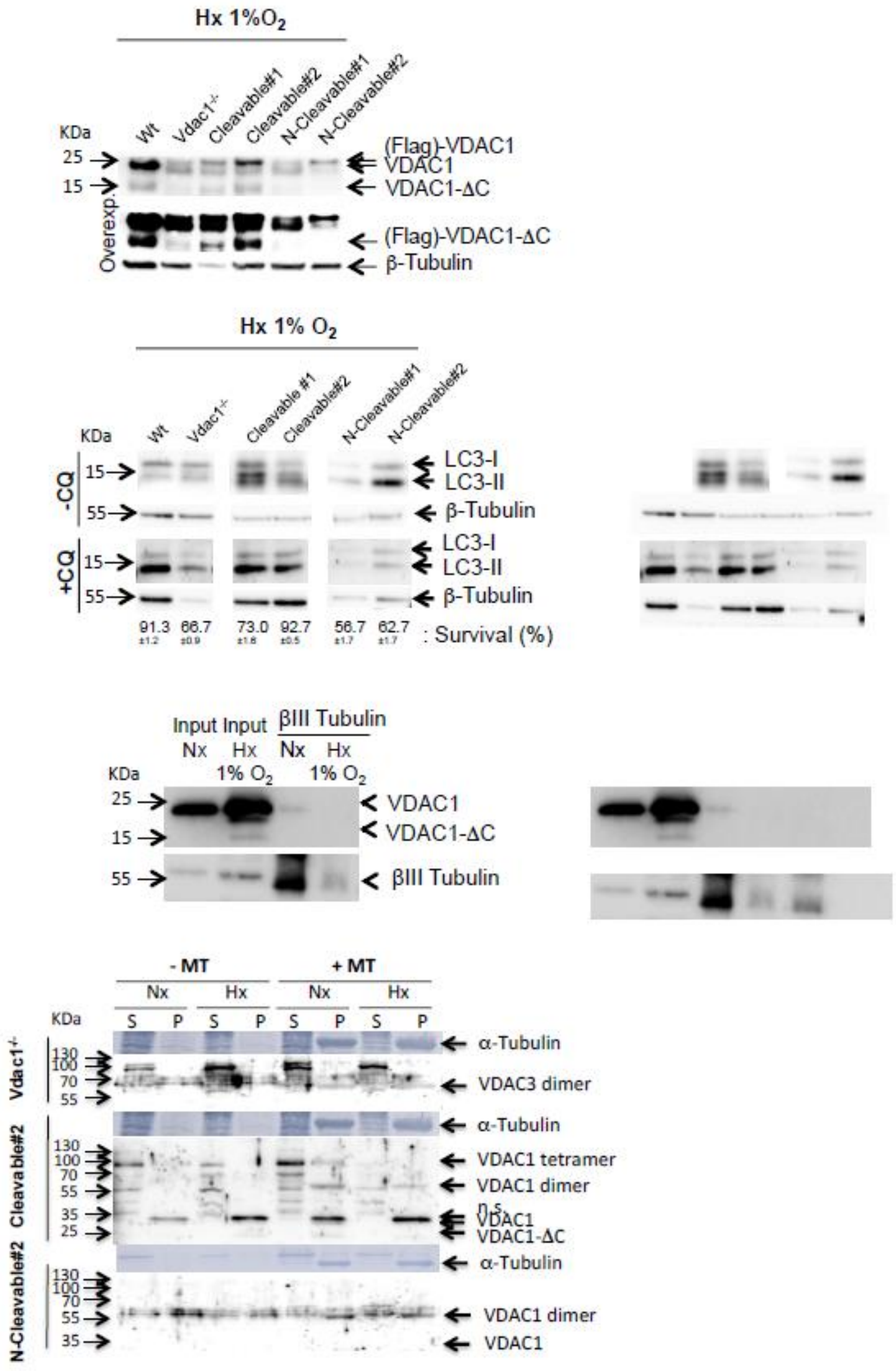

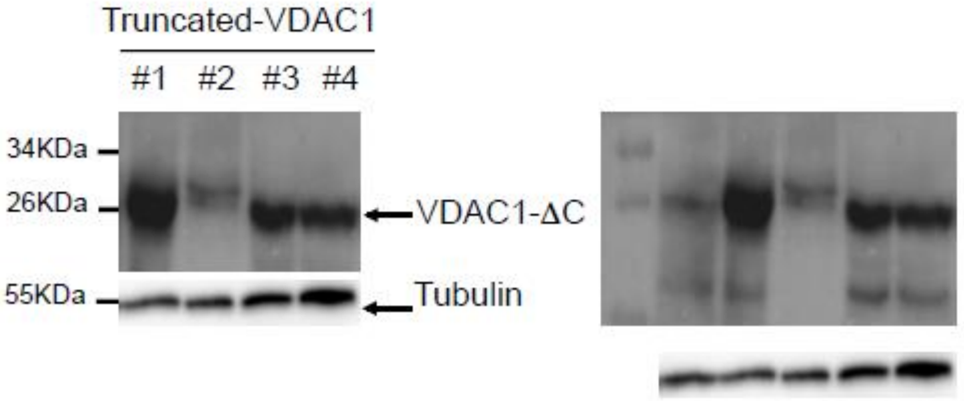

© 2020 by the author. Licensee MDPI, Basel, Switzerland. This article is an open access article distributed under the terms and conditions of the Creative Commons Attribution (CC BY) license (http://creativecommons.org/licenses/by/4.0/).
